# Characterization of an Insecticidal Toxin and Pathogenicity of *Pseudomonas taiwanensis* against Insects

**DOI:** 10.1371/journal.ppat.1004288

**Published:** 2014-08-21

**Authors:** Wen-Jen Chen, Feng-Chia Hsieh, Fu-Chiun Hsu, Yi-Fang Tasy, Je-Ruei Liu, Ming-Che Shih

**Affiliations:** 1 Institute of Biotechnology, National Taiwan University, Taipei, Taiwan; 2 Agricultural Biotechnology Research Center, Academia Sinica, Taipei, Taiwan; 3 Biopesticide Division, Taiwan Agricultural Chemicals and Toxic Substances Research Institute, Council of Agriculture, Taichung, Taiwan; 4 Institute of Molecular Biology, Academia Sinica, Taipei, Taiwan; Stanford University, United States of America

## Abstract

*Pseudomonas taiwanensis* is a broad-host-range entomopathogenic bacterium that exhibits insecticidal activity toward agricultural pests *Plutella xylostella*, *Spodoptera exigua*, *Spodoptera litura*, *Trichoplusia ni* and *Drosophila melanogaster*. Oral infection with different concentrations (OD = 0.5 to 2) of wild-type *P. taiwanensis* resulted in insect mortality rates that were not significantly different (92.7%, 96.4% and 94.5%). The TccC protein, a component of the toxin complex (Tc), plays an essential role in the insecticidal activity of *P. taiwanensis*. The Δ*tccC* mutant strain of *P. taiwanensis*, which has a knockout mutation in the *tccC* gene, only induced 42.2% mortality in *P. xylostella*, even at a high bacterial dose (OD = 2.0). TccC protein was cleaved into two fragments, an N-terminal fragment containing an Rhs-like domain and a C-terminal fragment containing a Glt symporter domain and a TraT domain, which might contribute to antioxidative stress activity and defense against macrophagosis, respectively. Interestingly, the primary structure of the C-terminal region of TccC in *P. taiwanensis* is unique among pathogens. Membrane localization of the C-terminal fragment of TccC was proven by flow cytometry. Sonicated pellets of *P. taiwanensis* Δ*tccC* strain had lower toxicity against the Sf9 insect cell line and *P. xylostella* larvae than the wild type. We also found that infection of Sf9 and LD652Y-5d cell lines with *P. taiwanensis* induced apoptotic cell death. Further, natural oral infection by *P. taiwanensis* triggered expression of host programmed cell death-related genes JNK-2 and caspase-3.

## Author Summary

Most entomopathogenic bacteria can produce toxin proteins and proliferate in the intestines of insects after natural oral ingestion. These bacteria can evade the systemic and local immune responses of insects. Here, we used insect larva of the diamond back moth (*Plutella xylostella*), a cruciferous crop pest to study the pathogenic mechanism of infection by novel bacterium *Psudomonas taiwanensis*. We examined how *P. taiwanensis* colonizes and escapes the immune response in the gut of *P. xylostella* and the mechanism of pathogenesis after oral ingestion of *P. taiwanensis*. Oral ingestion of *P. taiwanensis* induced severe damage in intestinal cells of *P. xylostella* and disrupted intestinal epithelial integrity. A toxin protein, the C component of insecticidal toxin protein complex (TccC) contributed to the pathogenicity of *P. taiwanensis* by thwarting oxidative stress and phagocytosis and inducing apoptosis in the gut cells of the host. Taken together our results shed light on the bacterial pathogenic processes in insect hosts, particularly the mechanism of pathogenesis of *P. taiwanensis*.

## Introduction

The pathogenic ability of many insecticidal bacteria depends on toxin complexes that consist of proteins encoded by gene clusters scattered around their genomes [1], [2]. Toxin complexes (Tcs) comprise three different classes of functional components, TcA, TcB and TcC, which display different insecticidal functions in several entomopathogenic bacteria [2]. However, the mechanism by which Tcs cause insect lethality remains largely unknown. Most studies have reported that full insecticidal activities require intact toxin complexes, which are formed by cross-linking of the A, B and C components [3]–[5]. In the bacterium *Photorhabdus luminescens*, toxicity and insecticidal activity of the A component (TcdA) was enhanced by co-expression of the B (TcdB) and C components (TccC) [4], [5]. When TcdA1 or TcdB2/TccC3 or TcdB2/TccC5 fusion proteins were used alone for infection of *Galleria mellonella* hemocytes, phagocytosis was ineffective. However, a combination of TcdA1 and TcdB2/TccC3 or TcdB2/TccC5 produced toxicity [5].

Nevertheless, the A component (TcdA) of *P. luminescences* W14 expressed at a high level in transgenic *Arabidopsis* is sufficient to cause high toxicity to *Manduca sexta* [6]. Lee et al. analyzed the 3-D structure of the A component TcA-like protein (XptA1) of *Xenorhabdus nematophila* PMFI296 and found that this protein formed a bottle-shaped tetrameric complex and bound to target cell membranes, and might be responsible for delivering the toxin complex [7]. Fragment analysis of TcdA showed that its N-terminus causes rearrangement of actin cytoskeleton and its C-terminus of coiled-coil domain promotes protein-protein interactions in mammalian tissue culture cells [4]. In the B component, the N-terminus of TcdB1 contains SpvB-like domain and C-terminus contains RCC1-like domains, which also causes actin contraction [4]. The SpvB domain encodes a mono-ADP-ribosyltransferase [8] and the RCC1-like domain mediates chromatin condensation [9]. In Tcs, the B component may interact with the A and C components and modify the C component [4]. Although TccC from *Xenorhabdus* alone has a high toxicity using a microsyringe injecting method against the wax moth *Galleria* [10], TccC recombinant protein isolated from *E.coli* expressing *Photorhabdus tccC* gene displays little oral activity alone [3], [4]. TcdA1/TcdB2/TccC3 fusion protein modifies threonine-148 of actin by ADP-ribosylation and induces actin clustering in *G. mellonella* hemocytes and HeLa cells [5]. However, TcdA1/TcdB2/TccC5 fusion protein modifies glutamine-61 and glutamine-63 of RhoA by ADP-ribosylation [5]. This suggests that different amino acid regions in TccC3 and TccC5 are responsible for their biological activities and diverse toxin components might derive different functional activities from variable compositions of domains and motifs in different entomopathoenic bacteria. Also, the possibility that a single component of the Tcs has oral toxicity against insets cannot be excluded.

Despite extensive studies on functions of the Tc components, little is known about the relationship between pathogenicity caused by Tc components and insect immune responses. Reactive oxygen species (ROS), anti-microbial peptides (AMPs), lysozymes, pattern recognition proteins, circulating recognition molecules and phagocytes are involved in the defense mechanisms of the insect immune system [11]. After oral ingestion, the local immune system is triggered in the intestinal tract cells of *Drosophila* to produce AMPs and ROS, important and complementary contributors to defense against ingested microbes [12], [13]. Pathogens have developed strategies to counteract host immune responses, evading the local immune system to promote pathogenicity [14]. *Pseudomonas entomophila*, which lacks a type III secretion system, develops multiple virulence factors to promote its pathogenicity [15]. Among these virulence factors, AprA metalloprotease plays an important role in fighting against the AMPs of *Drosophila* [14]. In addition, *P. entomophila* causes changes in expression levels in the genes that modulate the cytoskeleton components of the gut epithelium of the host through the JNK pathway [16]. Oxidative burst increases epithelium renewal and boosts the gut homeostasis system, which induces stem cell proliferation. In the *Drosophila* midgut, epithelium renewal is essential to defend against bacterial oral infection and is controlled by the immune response. The JNK (c-JNK NH_2_ terminal kinase) pathway is required for intestinal stem cells to maintain and proliferate after human pathogen *Pseudomonas aeruginosa* [17], [18] and insect pathogen *Pseudomonas entomophila* infection [19].

*Pseudomonas taiwanensis* is a novel Gram-negative bacterium isolated from soils that can grow on medium with shrimp shell powder as the sole carbon and nitrogen source [20]. Interestingly, *P. taiwanensis* displays high levels of extracellular chitinasae, chitosanase, and nattokinase activities under shrimp shell medium [21], [22]. *P. taiwanensis* also has a broad-host range of insecticidal activity against a Dipteran species (*Drosophila melanogaster*) and a number of Lepidopteran species (*Plutella xylostella*, *Spodoptera exigua*, and *Trichoplusia ni*). Recombinant TccC from *P. taiwanensis* alone caused mortality of *Drosophila* larvae, indicating that the TccC of *P. taiwanensis* has toxic properties independent of other components [23]. Comparison of amino acid sequences showed that the N-terminal fragments of different TccC components are highly conserved, while the C-terminal regions of TccC components are hypervariable [5], [24]. In this study, we investigated the mechanisms underlying the colonization of *P. taiwanensis* in the guts of insect larvae, its evasion of host immune response and its disruption of the intestinal barrier after oral ingestion. We analyzed the damage caused by *P. taiwanensis* and examined the signaling pathways triggered by *P. taiwanensis* in larval guts after oral infection. In addition, we characterized the function of the virulence factor TccC of *P. taiwanensis* and showed that the C terminus of TccC from *P.taiwanensis* might play an important role in its pathogenicity.

## Results

### Insecticidal activity of TccC of *P. taiwanensis* toward *P. xylostella*

**Figure 1:**
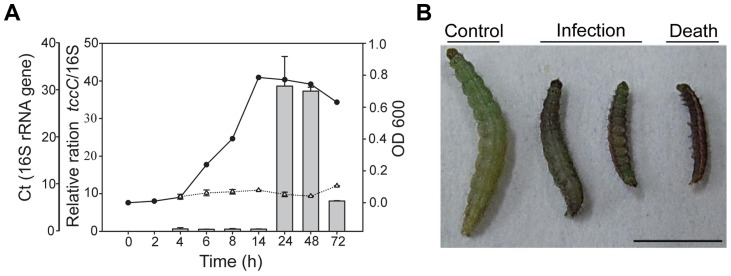
Relative expression levels of *tccC* and toxicity of *P. taiwanensis* to *P. xylostella* larvae. (A) The *tccC* expression levels in different growth phases (grey bar) of *P. taiwanensis* were analyzed by real-time PCR and compared with internal control 16S rRNA gene (white triangle). Growth curves of *P. taiwanensis* were measured by OD_600_ (black circle)_._ (B) *P. xylostella* larvae were infected after 3 days of oral ingestion by *P. taiwanensis*. Left, control (non-infection). Center, infection. Right, death. Larvae, three instar. Ingestion does: 50 µl OD = 1 cells/0.5×1 cm^2^ vegetable block. Scale bar = 0.5 cm.

In a previous study, the *tccC* gene from *P. taiwanensis* was overexpressed in *E. coli* and the recombinant TccC was able to increase the mortality in *Drosophila* larvae [23]. In addition to *Drosophila melanogaster*, we found that *P. taiwanensis* has insecticidal activity against a number of Lepidopteran species, including several vegetable pests *Plutella xylostella*, *Spodoptera exigua* and *Trichoplusia ni* (Table S1). Here, we investigated the *in vivo* insecticidal activities of the *P. taiwanensis* TccC against the Lepidopteran species *P. xylostella*. The expression level of TccC in *P. taiwanensis* was highest when bacterial cells reached the stationary phase (24 h) (Figure 1A). Therefore, we collected *P. taiwanensis* cells at this stage and determined their toxicity. The *P. taiwanensis* cells were orally administered to the *P. xylostella* larvae. The larvae in the treatment group exhibited slower growth and were melanized, dehydrated, and rigid in comparison with those in the control group (Figure 1).

We compared the amino acid sequences of several TccC-like proteins from different pathogens, and found that all of them had an N-terminal conserved RhsA-like domain and a C-terminal hypervariable fragment (Figure S1). Interestingly, the TccC of *P. taiwanensis* has a unique sodium/glutamate symporter-like domain and a TraT-like domain in the C-terminal region (Figure S1). In order to evaluate the function of the TccC protein, we generated an isogenic *tccC* gene knockout mutant, designated Δ*tccC*, of *P. taiwanensis* (Figure S2). Table 1 shows the mortality rates of *P. xylostella* larvae orally administered with whole cells or different cell fractions of wild-type or Δ*tccC P. taiwanensis*. The mortality of *P. xylostella* larvae infected with *P. taiwanensis* Δ*tccC* strain (OD = 2.0) was only 42.4% while those infected with wild-type *P. taiwanensis* was 94.5% (Table 1). We further prepared different cellular fractions of *P. taiwanensis* (Figure S3) and tested their effects on *P. xylostella* larvae. More than 50% of *P. xylostella* larvae infected with cell lysates, insoluble lysates (cell membranes and cell wall pellets) and extracellular supernatants of wild-type *P. taiwanensis* died at the end of the 5-day feeding period (Table 1). Moreover, the mortalities of *P. xylostella* larvae infected with cell lysates and insoluble pellets of *P. taiwanensis* Δ*tccC* were lower than those infected with wild-type lysates (Table 1). These results indicate that the insecticidal activity of *P. taiwanenesis* might be attributable, at least in part, to the TccC.

**Table 1:** *P. xylostella* mortality after oral infection with *P. taiwanensis* wild-type and Δ*tccC* strains or their cell lysate, sonicated pellet, sonicated supernatant, or culture supernatant on day 5.

Treatment^a^	% Treated mortality (*n*)^b^	P value (two tailed)^c^
**Control** d	1.8% (1/55)	P<0.05
**Whole cells of ** ***P. taiwanensis*** e		
**Wild-type strain**		
OD = 0.5	92.7% (51/55)	P<0.05
OD = 1.0	96.4% (53/55)	P<0.05
OD = 2.0	94.5% (52/55)	P<0.05
**Δ** ***tccC*** ** mutant strain**		
OD = 2.0	42.4%(14/33)	P<0.05
**Crude extract of ** ***P. taiwanensis*** f		
**Wild-type strain**		
Cell lysates	67.3% (33/49)	P<0.05
Insoluble lysates	50.0% (24/48)	P<0.05
Soluble lysates	31.3% (15/48)	P<0.05
Secretory proteins	65.9% (29/44)	P<0.05
**Δ** ***tccC*** ** mutant strain**		
Cell lysates	45.6% (21/46)	P<0.05
Insoluble lysates	25.0% (11/44)	P<0.05
Soluble lysates	32.7% (16/49)	P<0.05
Secretory proteins	64.3% (27/42)	P<0.05

^*a*^ *P. taiwanensis* wild-type, Δ*tccC* mutant strains, and their various proteins fractions were fed to three instar of healthy larvae.

^*b*^Mortality is the percentage of larvae death. n is the sample size of the treated groups. The data were collected on day 5.

^*c*^The two tail student *t*-test was used to elucidate statistical significance. Each treatment was repeated three times.

^*d*^Similarity with *b*, n is the sample size of the negative control PBS-treated group.

^*e*^Ingestion dose: 50 µl OD = 0.5, 1, 2 cells/0.5×1 cm^2^ vegetable block.

^*f*^Ingestion dose: The crude extract contained 300 ng of proteins.

**Figure 2:**
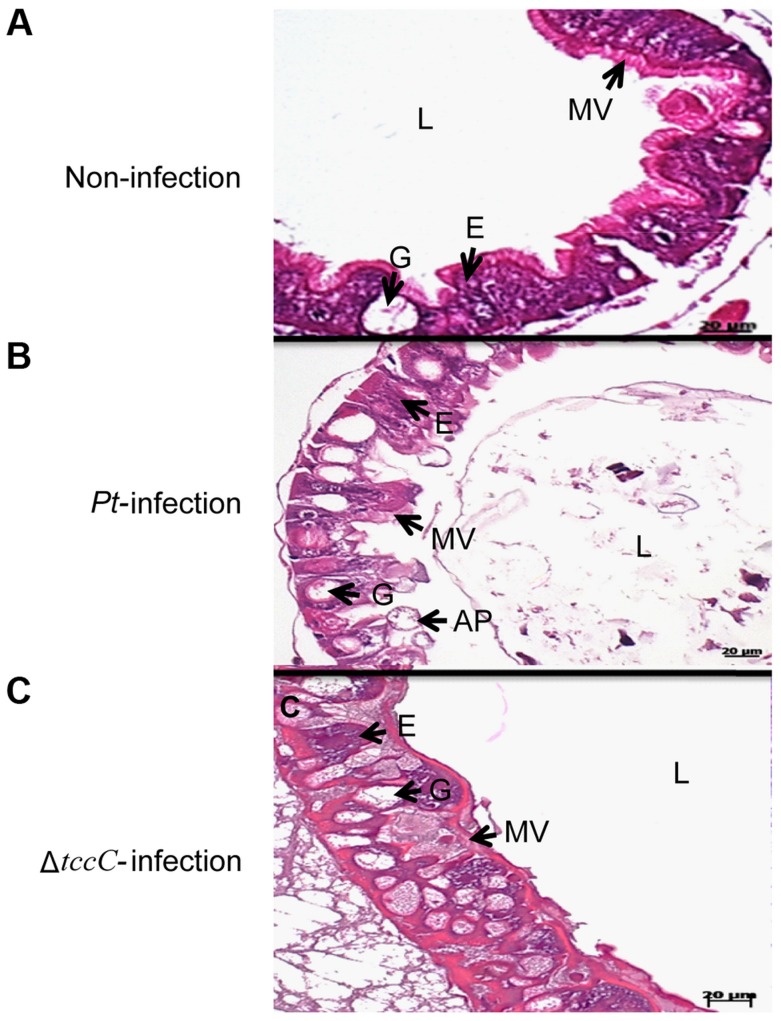
TccC of *P. taiwanensis* contributes to damage to the midgut of *P. xylostella* larvae. Histological tissue of midgut sections were collected with (A) non-infection or (B) 48 h after oral infection by wild-type or (C) *tccC* mutant *P. taiwanensis*. In non-infection larva, the larval stage epithelium presents enterocytes (E, commonly called columnar cells), goblets cells (G), microvilli (MV). After 48-h oral infection with *P. taiwanensis* considerable damage to gut cells such as apical protrusion (AP), cell lysis, and abnormal microvilli can be seen. Although *tccC* mutant also causes abnormal microvilli, infection with *tccC* mutant did not result in any apical protrusion or cell lysis. In surviving larva, the damaged gut induced stem cell proliferation and differentiation and promoted gut cell renewal, resulting in *tccC* mutant-induced morphological alterations in goblet cells that are not seen in non-infected or wild-type *P. taiwanensis* infection insects. Scale bar = 20 µm.

Infection of Lepidopteran larvae with toxins, bacteria or viruses caused the appearance of apical protrusion and protrusion ruptures in the damaged enterocytes [25]. Therefore, we performed histological analyses to assess the effect of *P. taiwanensis* infection on the intestinal tracts of *P. xylostella*. The ultrastructure of the midgut of *P. xylostella* larva showed that oral infection with *P. taiwanensis* had a strong impact on gut cells (Figure 2). After infection with *P. taiwanensis* for 48 h, apical protrusion of enterocytes, abnormal microvilli and cell lysis were induced in the guts in *P. xylostella* (Figure 2B compared with 2A) indicating that *P. taiwanensis* infection caused serious injury to the midgut epithelial cells, which could not be repaired in the homeostatic process and finally caused the death of the host. Similarly, ultrastructure sections of *P. xylostella* larvae that ingested 100 ng toxin complex (Tc)/cm^2^ food, showed columnar cells in the guts containing many vesicle-like structures [26]. In contrast, ingestion of the Δ*tccC* mutant only resulted in abnormal microvilli in *P. xylostella* intestinal tracts, without any apical protrusions or cell lysis (Figure 2C).

Damage to the gut can induce stem cells to proliferate and differentiate to replace the damaged cells, producing a higher number of goblet cells with a larger size than the control group [25], [27], [28]. Homeostasis of gut cells, through which damaged cells are replaced with newly differentiated enterocytes and goblet cells, is very important during infection [18], [25]. Lethal pathogens are capable of overcoming host immune defenses, and eventually blocking gut homeostasis [17]. Of particular interest, in this study, we observed that oral infection of *P. xylostella* with *P. taiwanensis* Δ*tccC* resulted in a greater number of goblet cells in the midgut system (Figure 2C) as compared with the non-infected or wild-type *P. taiwanensis*-infected *P. xylostella* (Figure 2A and 2B) indicating that only infection with Δ*tccC*, but not the wild-type, could induce the differentiation of damaged cells and the formation of many goblets in the midgut system. On the other hand, we also found that enlargement of the goblet cavity did not occur in Δ*tccC* infection (Figure 2C). It is likely that over-proliferation of differentiated goblet cells replaced damaged gut cells when *P. xylostella* was infected by the Δ*tccC* mutant strain. Wild-type *P. taiwanensis*, however, displayed severe damage, inducing collapse of the epithelial layer, enlargement of goblet cells, and rupture of apical protrusion (Figure 2B). This suggests that the toxicity of *P. taiwanensis* Δ*tccC* was lower than that of the wild-type strain, and the midgut epithelial cells could be repaired (Figure 2C).

**Figure 3:**
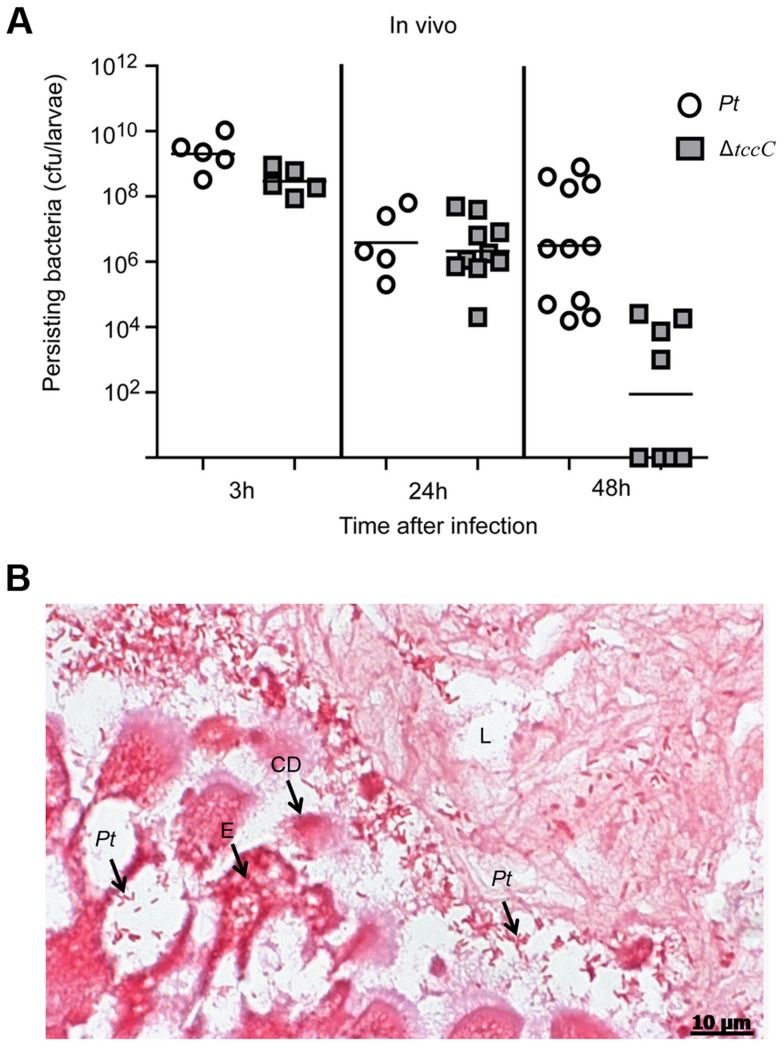
TccC is instrumental in *P. taiwanensis* persistence in the gut of *P. xylostella* and in invasion of the epithelium cells. (A) Comparison of colonization through bacteria persistence in *P. xylostella* larvae. After oral infection by *P. taiwanensis* wild-type strain and *tccC* mutant strain, the numbers of colony-forming-units (cfu) were calculated at different time points. (B) Gram stain of *P. xylostella* midgut tissue section was observed after oral infection by *P. taiwanensis* for 48 h. The epithelial cells were invaded and lysed by *P. taiwanensis*. At 48 h, *P. taiwanensis* crossed the epithelium barrier of midgut into the cells. *Pt*, *P. taiwanensis*; CD, cell debris; E, enterocytes; L, lumen. Scale Bar = 10 µm.

The colonization and invasion of midgut epithelial cells of *P. xylostella* by *P. taiwanensis* were further confirmed by bacterial quantification and histological examination. After oral infection for 48 h, the bacterial counts of *P. taiwanensis* Δ*tccC* were lower than those of wild-type strain in the midgut of *P. xylostella* (Figure 3A). In addition, the midgut epithelial cells were seriously disrupted by wild-type *P. taiwanensis* after oral infection for 48 h (Figure 3B).

**Figure 4:**
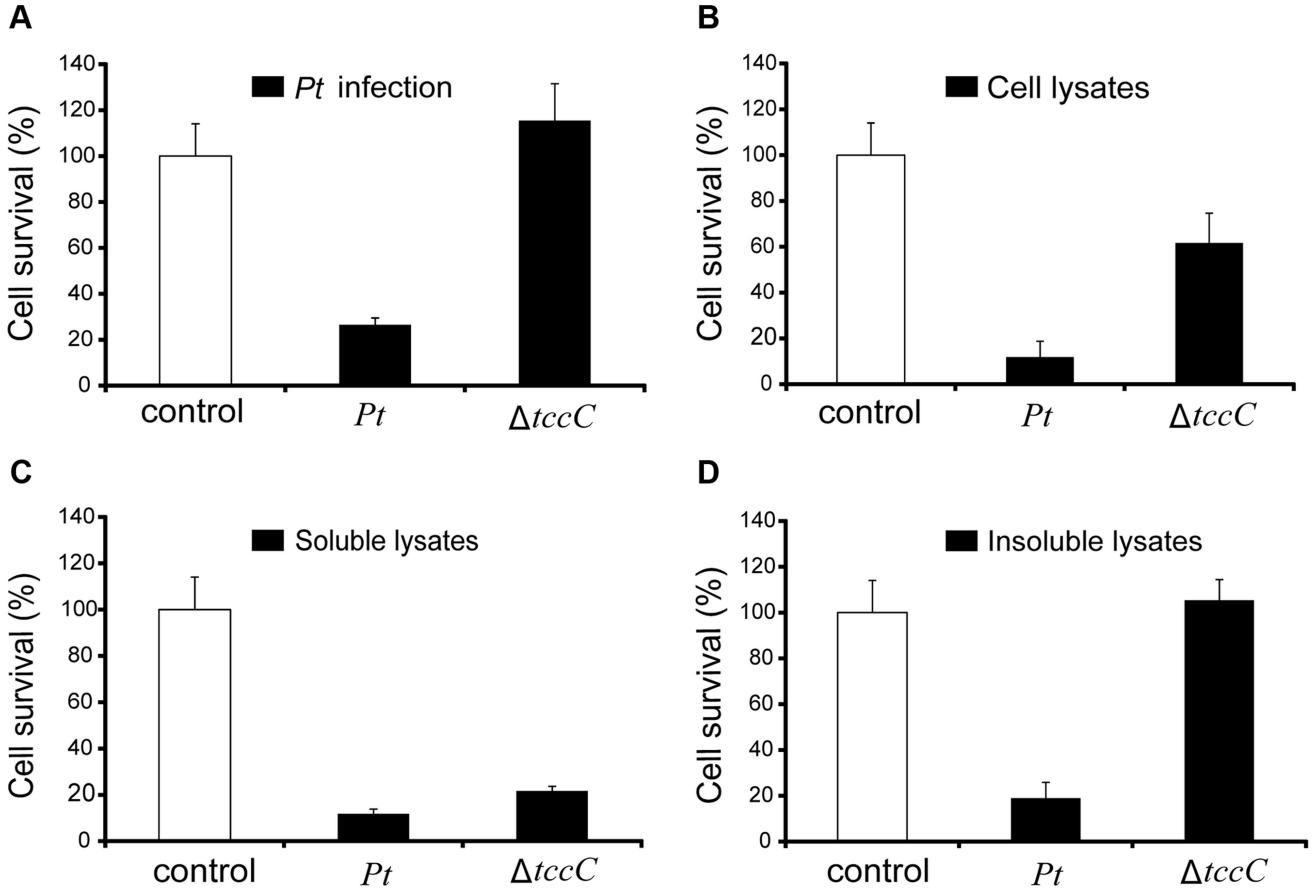
Toxicity of *P. taiwanensis* and various cell fractions on *Spodoptera frugiperda* Sf9 insect cells. (A) Survival rates of Sf9 cells after infection of *P. taiwanensis* wild-type and Δ*tccC* (MOI = 1000) and protein fractions (10 µg/ml) derived from (B) cell lysates, (C) soluble lysates and (D) insoluble lysates of *P. taiwanensis*. Every well in a 96-well plate contained 5000 Sf9 cells. The results are obtained by XTT proliferation assay *P. taiwanensis* infection or protein treatment for 72 h.

The insecticidal activity of the TccC was further confirmed by treatment of Sf9 insect cells with different *P. taiwanensis* cell fractions (Figure 4). The survival rates of Sf9 insect cells exposed to the intact cells (*P. taiwanensis* alive), cell lysate (total proteins), soluble lysate (cytosolic proteins) and insoluble lysate (cell wall and cell membrane) of wild-type *P. taiwanensis* were significantly lower than those exposed to PBS buffer (Figure 4A–D). On the other hand, the survival rates of Sf9 insect cells exposed to the intact cells or cell wall pellets of *P. taiwanensis* Δ*tccC* were not significantly different from those exposed to PBS buffer, only those exposed to the cell lysates or soluble lysate of *P. taiwanensis* Δ*tccC* were significantly decreased. Since *P. taiwanensis* Δ*tccC* did not express TccC, it was likely that some other virulence factors were present in the cell lysates of *P. taiwanensis* Δ*tccC*. Furthermore, active phagocytosis was found in Sf9 viable cells, a characteristic phenomenon during *in vivo* apoptosis but uncommon for *in vitro* cultures. Sf9 cells are phagocytic and contain unusually high numbers of phagosomes, particularly after glucose depletion [29]. In the early infection stage (after incubation for 1 h), RFP-labeled *P. taiwanensis* was phagocytosed by Sf9 cells (Figure S4A). After incubation for 3 h, lysis of Sf9 cells infected with *P. taiwanensis* was observed, as compared with no lysis in non-infected cells (Figure S4B and S4C).

### Induction of apoptotic cell death by TccC of *P. taiwanensis*

**Figure 5:**
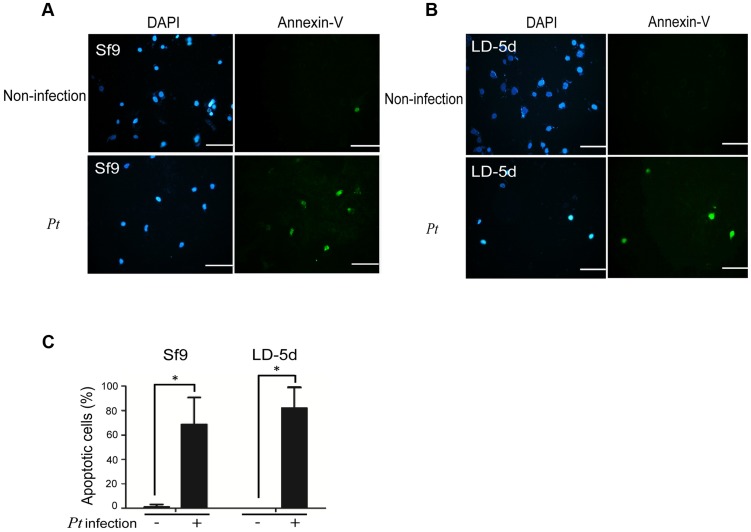
Apoptosis-induced by *P. taiwanensis* infection in Sf9 and LD-5d cell lines. After *P. taiwanensis* (MOI = 1000) infection for 10 h, the insect cell lines Sf9 (A) and LD-5d (B) were stained with Annexin V-FITC and DAPI staining. *P. taiwanensis* can induce apoptosis in Sf9 and LD-5d cells after infection for 10 h. (C) The frequencies of apoptopic cells in (A) and (B) were quantified by calculating the ratio of Sf9 and LD-5d to DAPI positive numbers. Scale bar = 20 µm.

**Figure 6:**
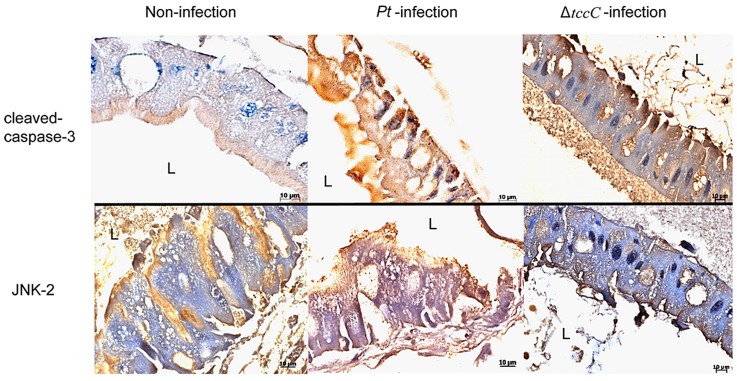
Immunohistochemical analysis of cleaved-caspase-3 and JNK-2 protein expression level in the gut tissue of *P. xylostella* larva. Expression level of caspase-3 was detected by immunohistochemical analysis using cleaved-caspase-3 or JNK-2 antibodies. Samples of larvae were collected after oral infection for 48 h by *P. taiwanensis* wild-type or Δ*tccC* strain.

In a previous study, both the JNK and JAK-STAT pathways and several cell proliferation genes of adult *Drosophila* were significantly induced after oral infection with *P. entomophila* [16]. The JNK pathway was activated in apoptotic or proliferative cells [30]. To determine whether *P. taiwanensis* infection induces apoptosis in Lepidopteran Sf-9 and LD-5d cells, we used Annexin V-FITC to stain for apoptotic cells and DAPI staining to determine total cell numbers. Apoptosis was detected in Lepidopteran Sf-9 and LD-5d cells after 10 h of infection with *P. taiwanensis* (Figure 5A and 5B) and significantly higher mortality rates were observed than in the non-infection control (Figure 5C). Furthermore, the JNK pathway of the gut epithelial cells of *P. xylostella* larvae was triggered by *P. taiwanensis* infection (Figure 6). In addition to the JNK pathway, we also examined the expression of the caspase genes, which can also induce apoptotic cell death [31]. After 48-h oral infection with *P. taiwanensis*, the expression level of cleaved-caspase-3 was increased in the midgut cells (Figure 6). The expression levels of JNK-2 and cleaved-caspase-3 in *P. xylostella* larva infected with *P. taiwanensis* Δ*tccC* were lower (Figure 5) than in the wild-type strain of *P. taiwanensis*, indicating that TccC might induce apoptosis and play an important role in cell death of the gut epithelial cells of *P. xylostella* larvae.

### Effect of TccC on the antioxidant response of *P. taiwanensis*

The digestive tracts of healthy insects are protected against bacterial disruption by an intact gut epithelial barrier and the host immune defense system. In the insect gut system, antimicrobial peptides (AMPs) and reactive oxygen species (ROS) are important elements of the defense system against invading pathogens [11]. In order to overcome the attack of AMPs, *P. entomophilas* secretes an abundant protease (AprA) that degrades AMPs [14]. We analyzed the protease and antioxidant responses of *P. taiwanensis* strains to evaluate their resistance against the insect gut immune system. At the stationary phase of bacterial growth, *P. taiwanensis* secreted large amounts of proteases (Figure S5) and showed high antioxidant response (Figure S6). Interestingly, the antioxidant response of *P. taiwanensis* Δ*tccC* was significantly lower than that of wild-type *P. taiwanensis* (Figure S6), indicating that the antioxidant response of *P. taiwanenesis* might be directly or indirectly regulated by the TccC.

**Figure 7:**
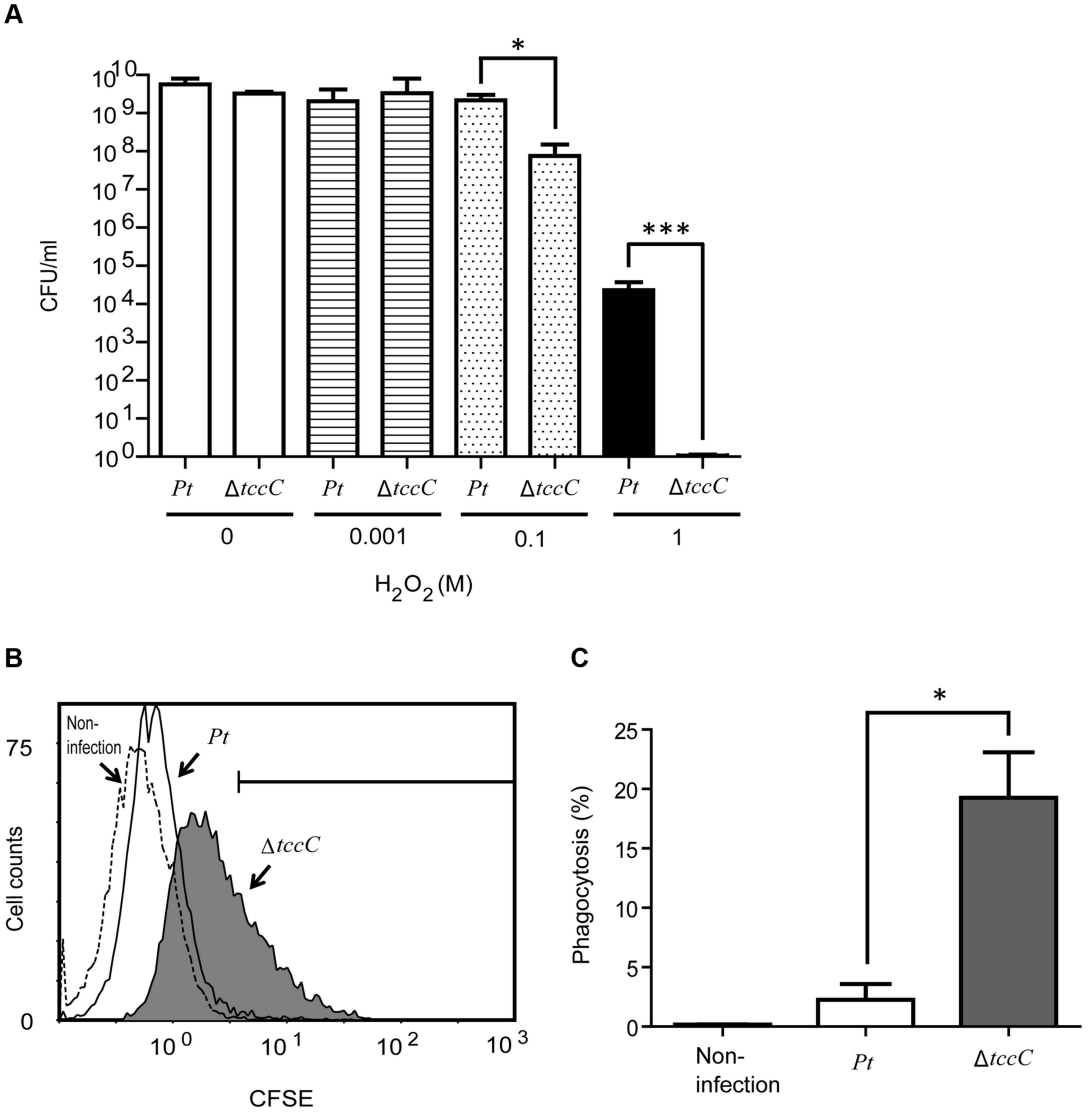
TccC contributes to increases in oxidant stress defense and inhibits phagocytosis. (A) Antioxidant response was measured by different H_2_O_2_ concentration treatments. Wild-type and *tccC* mutant of *P. taiwanensis* were exposed to different concentrations of H_2_O_2_ for 3 h and surviving cells were detected by CFU. (B–C) Phagocytosis of CFSE-stained wild-type or *tccC* mutant of *P. taiwanensis* by macrophages was detected. CFSE-stained wild-type or *tccC* mutant of *P. taiwanensis* incubated with macrophage (MOI = 1000) for 30 min and (B) following analysis of phagocytosis and (C) quantification by flow cytometry. Untreated macrophage cells were used as a negative control.

In order to confirm the involvement of the TccC in antioxidant response, wild-type and Δ*tccC P. taiwenansis* were exposed to different concentrations of hydrogen peroxide and the bacterial counts were determined. The results showed that wild-type *P. taiwenansis* had a higher survival rate than Δ*tccC* (Figure 7A), demonstrating that TccC also played a role in the protection of bacterial cells against ROS. ROS induced greater damage in the *tccC* mutant at high concentrations of H_2_O_2_ treatment (Figure 7A). The *P. taiwanensis* TccC protein contains a sodium/glutamate symporter Glts–like domain in its C-terminal, which might function in glutamate transport. To compare the glutamate uptake activity of wild-type and ΔtccC mutant, *P. taiwanensis* cells were cultured in medium containing 250 µM of ^15^N-L-glutamate for 4 hours. Uptake activity of ^15^N-L-glutamate was defined as enrichment of ^15^N content in *P. taiwanensis* cells. The results showed that glutamate uptake activity was much lower in ΔtccC than in the wild-type (Figure S7), which is consistent with the hypothesis that TccC has glutamate import activity. Since L-glutamate can be converted to glutathione, TccC might play a role in defense against ROS attack and maintain the intracellular redox potential in *P. taiwanensis*. We, therefore, next determined whether *P. taiwanensis* possesses the ability to degrade hydrogen peroxide (H_2_O_2_). We found that 1 mM H_2_O_2_ was quickly degraded after incubation with wild-type *P. taiwanensis* for 2 min. In contrast, it took 15 min to completely decompose when incubated with *tccC* mutant (Figure S6). Together, our results suggest that wild-type *P. taiwanensis* has higher H_2_O_2_ detoxification activity, and is, therefore, more resistant to ROS attack generated by the host immune response than the *tccC* mutant.

### Antiphagocytic activity of TccC

To evaluate the antiphagocytic activity of TccC, we performed a phagocytosis assay in which wild-type and Δ*tccC P. taiwanensis* cells were fluorescent-labeled with CFSE and then incubated with mouse macrophage cells. As seen in the scatter plot in Figure 7B, macrophage cells incubated with fluorescent-labeled *P. taiwanensis* Δ*tccC* for 30 min showed a shift in the peak position toward higher fluorescence intensity, indicating that the amount of phagocytized Δ*tccC* was larger than that of phagocytized wild-type *P. taiwanensis*. To substantiate the findings of the scatter plot analysis, the percentage of phagocytized *P. taiwanensis* was calculated (Figure 7C). The mouse macrophages engulfed fewer wild-type cells than the Δ*tccC* cells, suggesting that wild-type *P. taiwanensis* possessed antiphagocytic activity that might be partly attributable to TccC. We also analyzed the cytotoxicity of *P. taiwanensis* wild-type and Δ*tccC* toward mouse macrophages and found that the survival rate of mouse marcophages in the presence of the wild-type was not different from that in the presence of Δ*tccC*, suggesting that *P. taiwanensis* does not have a cytotoxic effect on mouse macrophages (Figure S8).

### Processing and location of TccC *in vivo*

**Figure 8:**
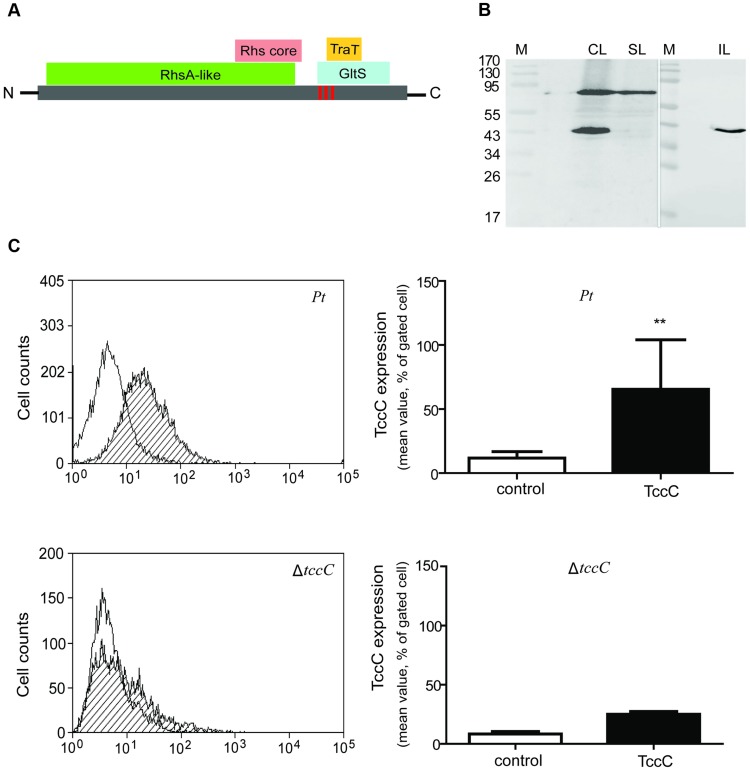
TccC processing and locations of fragments in *P. taiwanensis*. (A) According to the predicted protein domains, TccC possesses a highly conserve RhsA protein (11-673) in the N-terminus and Rhs repeat-associated core domain (600-680), sodium/glutamate symporter-like (726-825), and TraT complement resistance-like proteins (736-781) in the C-terminus. The vertical line indicates three transmembrane fragments at the C-terminal hypervariable region ( IIYVLMSVVLEALATTIGMAGGLLGGAAGGAIGGAVGGVIANVPGAAVGATWGASVGGLVG ). The amino acid sequence of the transmembrane helix was predicted by Philius transmembrane prediction server. (B) TccC fragments were detected in different locations in *P. taiwanensis* by western blotting using TccC antibody. Cell lysate (CL), soluble lysate (SL), and insoluble lysate (IL) to confirm TccC location was obtained according to the experimental steps outlined in Figure S3. (C) TccC was measured by flow cytometry after staining with TccC-FITC antibody. Wild-type and Δ*tccC* were compared with the non-stained control of *P. taiwanensis*.

Based on Pfam domain prediction, TccC is predicted to possess an RhsA domain (11-673), an Rhs repeat-associated core (600-680), and sodium/glutamate symporter-like (726-825) and TraT complement resistance-like domains (736-781). In addition, three transmembrane regions (718-742, 744-758, 760-778) were predicted at the C-terminal region (Figure 8A, vertical red bars). Western blot analyses were performed to determine the subcellular localization of the TccC protein in *P. taiwanensis* (Figure 8B). Three cellular fractions were prepared according to the method outlined in Figure S3. Surprisingly, two protein bands were detected in the total cellular protein fraction, a ∼70 kD and a ∼40 kD bands, representing a processed form of TccC protein (Figure 8B, lane CL). In the soluble protein fraction, only the ∼70 kD band was detected (lane SL), whereas in the insoluble pellet fraction that contained cell wall and membrane proteins only, the processed ∼40 kD band was detected (lane IL). This suggests that TccC protein was processed when it was inserted into the membrane of *P. taiwanensis* cells. Because the C-terminal of the TccC contains three transmembrane regions, we suspect that the C-terminal domain of TcCC was integrated into the membrane.

We have observed that the recombinant TccC protein also was similarly processed in *E. coil* expression system. To further characterize the cleavage process, TccC with 6xHis-tag was cloned into a broad host range vector pCPP30, and overexpressed in *P. taiwanensis* and *E.coli* (BL21) (Figure S9). The His-tagged TccC proteins were purified using a nickel ion column. Western blot analysis showed that processed forms of TccC proteins with similar molecular weight were purified from both *E. coli* and *P. taiwanensis* (Figure S9). This result suggests that the TccC has a similar cleavage site in *E.coli* and *P. taiwanensis*.

To test whether the TccC was indeed integrated into cell membrane, the TccC was labeled with FITC to trace the outer membrane fraction by staining with TccC-FITC antibody. Flow cytometry analysis showed that the fluorescence signal of TccC on the cell surface of *P. taiwanensis* had significantly higher density than the non-stained control (Figure 8C). In contrast, no significant fluorescence density was detected in the *tccC* mutant.

## Discussion

Although toxin complex (Tc) genes that encode insect toxins are found in many entomopathogenic bacteria, the mechanisms of action of these toxin complexes are not well understood. In our previous study, we found that oral ingestion of the purified recombinant C component of the toxin complex TccC-like protein from *P. taiwanensis* caused high mortality in *Drosophila* [23]. In this study, we demonstrate that *tccC* mutant has lower toxicity toward larvae of the agricultural pest *P. xylostella* than the wild-type (Table 1). However, *tccC* mutant still induces a 42.4% mortality rate. Therefore, in addition to TccC as an insecticidal factor, *P. taiwanensis* also triggers others virulence factors or cofactors to produce the toxic effect. From our unpublished whole genome sequencing data, we found many virulence-related factors, such as other components of insecticidal toxin complexes (*tccA*, *tccB*, *tcdB*), lipases, proteases, chitinase, nonribosomal peptides and repeat-in-toxin (RTX), which might have toxic effects on insects.

In the genome of *P. taiwanensis* there are two homologus *tccC* genes located in different gene clusters. TccC (846 aa) and TccC2 (896 aa) have 57% amino acid sequence identity in the RhsA domain (Figure S1), but have little similarity in other regions. TccC has predicted Glt and TraT domains in the C-terminus, while TccC2 has no predicted domains in the same region. We, therefore, propose that TccC and TccC2 have different functions in *P. taiwanensis*. TccC is induced in the stationary phase in *P. taiwanensis* (Figure 1A), and like the toxin complex genes of *Clostridium botulinum* is upregulated in the early stationary phase [15], [32], [33]. In *P. entomophila*, which also infects *Drosophila* [15], the GacS/GacA system regulates secondary metabolite formation, and influences sigma factor σ^s^ accumulation to respond to stress of *Pseudomonas* spp. in the stationary phase [34]. Virulence and pathogenicity are also regulated by the GacS/GacA two-component system [35]. Based on the similar expression patterns of these toxin genes, we propose that TccC is also regulated by GacS/GacA in *P. taiwanensis*.

Histological tissues of midgut sections of *P. xylostella* displayed severe damage caused by the ingestion of wild-type *P. taiwanensis*, but ingestion of *tccC* mutant did not cause dramatic damage (Figure 2). The *tccC* mutant induced *P. xylostella* larvae gut stem cell proliferation and cell renewal (Figure 2C compared to 2A). Conversely, wild-type *P. taiwanensis* disrupted gut cell renewal (Figure 2B compared to 2A) and entered into gut cells (Figure 3B). These results indicate that *P. taiwanensis* can evade insect immune responses and colonize in the gut (Figure 3). *Drosophila melanogaster* produces antimicrobial peptides (AMPs), reactive oxygen species (ROS) and hemocytes in local and systemic responses to defend against pathogens [11], [14], [36]. Pathogens can degrade AMPs by zinc metalloprotease (AprA), protect cells from ROS-induced damage by antioxidant enzymes, and inhibit phagocytosis by hemocytes [11], [14], [36]. Because *Drosophila* immune responses are shared with other higher organisms [36]–[38], we used the information gained from the *Drosophila* studies to investigate the interaction between agricultural pest *P. xylostella* and *P. taiwanensis*. Our results showed that *P. taiwanensis* is involved in resistance to a burst of high H_2_O_2_ in the environment (Figure 7A) and is capable of degrading H_2_O_2_ (Figure S6). *P. taiwanensis* also produces proteinase activity in the stationary phase (Figure S5). In phagocytosis, *P. taiwanensis*-RFP labeled cells were engulfed by Sf9 cells to form phagosomes, resulting in the lysis of Sf9 cells (Figure S4). These mechanisms together enable *P. taiwanensis* to effectively colonize in the gut of *P. xylostella* larvae. In an interaction between *P. taiwanensis* and mouse macrophages, *P. taiwanensis* displayed antiphagocytosis activity (Figure 7B and 7C). However, *P. taiwanensis* did not cause mouse macrophage death (Figure S8). Similar to entomopathogenic *P. entomophila* [15], *P. taiwanensis* did not infect macrophages because it lacks a type III secretion system (unpublished whole genome database).

The Tc has been well characterized from the insect pathogen *P. luminescens*, and the TcC component that has biological activity in the C-terminal hypervariable region have been identified as ADP-ribosyltransfeases [39]. A previous study showed that TcC-like (TccC) protein may be cleaved and coexpressed with TcB (TcdB) by an unclear mechanisms [4]. Recently, Aktories and colleagues showed that TcC was autoproteolytically cleaved and secreted the C-terminal region into host cells by the TcA channel of the tripartite Tc toxin complex [39]. However, in the *P. taiwanensis* system, we found that TccC was autoproteolytically cleaved and the cleaved-C-terminus was integrated into the cell membrane (Figure 8B and S9). This result is consistent with the observation that the C-terminus of TccC consists of a transmembrane GltS-like domain and a TraT-like domain (Figure 8A). The Tc mechanism seems to be very different in *P. taiwanensis* and *P. luminescens*. The biological activity of TccC depends on C-terminal functional regions. Therefore, there may be functional differences in Tcs across pathogens that correlate with the differences in the amino acid sequences in the hypervariable regions of TccCs (Figure S1).

Analysis of the *tccC* mutant indicated that TccC, which is associated with defense against local immune response, significantly contributes to the colonization of *P. taiwanensis* in the gut (Figure 3A). Considerably less damage was seen in the intestinal tract after ingestion of the *tccC* mutant. The *gltS* gene encodes the sodium-dependent glutamate symporter which transporters L-glutamate into bacterial cells [40], [41]. Nitrogen metabolism of L-gultamate plays an important role in the colonization and pathogenicity of some bacteria such as *Streptococcus pneumonia*, *Helicobacter pylori* and *Neisseria meningitidis* [42]–[44]. In the L-glutamate uptake system of *N. meningitidis*, L-glutamate is converted into glutathione, which can detoxify ROS produced by polymorphonuclear neutrophil leucocytes [44]. In the insect gut system, ROS is an important element in the defense against invading pathogens [11]. In an in vitro analysis, we showed that the *tccC* mutant had less H_2_O_2_ detoxification activity, which might be attributed to the function of the GltS-like domain (Figure 7A). Conversely, we propose that the TraT-like domain in the C-terminus of TccC might have other functions. TraT is an outer membrane protein that mediates the barrier function, which excludes toxic compounds such as bile salts, lysophosphatides, lysozymes, phosphlipases and proteases in the environments from entering cells [45], [46]. TraT can also inhibit phagocytosis by macrophages [47]. The *tccC* mutant of *P. taiwanensis* increased phagocytosis by mouse macrophage cells (Figure 7). However, we cannot exclude the possibility that reduced toxicity of the *tccC* mutant *per se* resulted in enhanced phagocytosis. Previous studies showed that *P. luminescens* inhibited phagocytosis by utilizing the toxin complex rather than a single component [5], [48]. Therefore, it is likely that the Tc complex is not formed in the *tccC* mutant, affecting the antiphagocytosis activity of the mutant. Outermembrane proteins and lipoproteins are known to be involved in host-pathogen interactions, such as adhesion and permeability barrier, maintaining intracellular physiological functions, and promoting host immune responses and barrier disruption by pathogens [49], [50]. Among the Tcc genes with known sequences, only the C- terminal of TccC from *P. taiwanensis* has Glts and TraTdomians with potential permease functions. These permease activities might contribute to the insecticidal activity after oral infection by *P. taiwanensis*.

A number of studies have demonstrated that Tc toxin complexes are involved in pathogenicity in insects; however, none has shown whether signal transduction pathways are triggered by Tc toxin complexes or by a single component. Our results showed that *P. taiwanensis* triggers the JNK and caspase pathways in the gut system, which mediate inflammation and apoptosis of damaged cells, respectively (Figure 6). The *tccC* mutant did not induce the JNK and caspase pathways in gut cells, suggesting that TccC plays a role in triggering the cell homeostasis and apoptosis signaling pathways. Consistently, infection of *P. taiwanensis* induced apoptosis in insect cell lines (Figure 5). Histological analysis of the midgut showed that the epithelium cells sustained less damage and displayed cell renewal after infection by the *tccC* mutant (Figure 2C). These data demonstrate that TccC has a significant effect on the *P. taiwanensis* and *P. xylostella* interaction and contributes to cell death mediated by JNK- and caspase-depend pathways. Cleaved-caspase-3 is the active form of caspase-3 in damaged cells and is regarded as an apoptotic initiator in mammalian cells. Insects like *Drosophila* and *Choristoneura fumiferana* are also known to have a similar mechanism [31], [51]. In this study, our IHC analysis proved that cleaved-caspase-3 accumulates in the damaged gut cells of *P. xylostella* after infection by *P. taiwanensis* and thus induces apoptosis. Previously, many studies have focused on using recombinant proteins to examine the toxicity of Tcs, but the mechanism by which Tc complexes work and the host cells respond are still poorly understood. The results presented here demonstrate that the TccC in *P. taiwanensis* can induce host damage by triggering signal transduction of the apoptosis pathway after oral infection.

## Materials and Methods

### Bacterial strains, culture condition, and antibiotics

*P. taiwanensis* BCRC 17751 [23] was used as the entomopathogenic species. *Escherichia coli* DH5α was used in all construction experiments. *E. coli* S17-1 [52] was used for biparental mating with *P. taiwanensis*, and *E.coli* BL21 was used to express recombinant protein. *P. taiwanensis* and *E. coli* were grown in Luria-Bertani (LB) broth or on an agar plate. *P. taiwanensis* cultures were grown at 30°C and *E. coli* cultures were grown at 37°C. Antibiotics were applied at the following concentrations: rifampicin (34 µg/ml), ampicillin (100 µg/ml), and spectinomycin (100 µg/ml) for *P. taiwanensis* wild-type cultured media; and kanamycin (30 µg/ml), tetracycline (20 µg/ml) for *P. taiwanensis* mutant strain and overexpression strain, respectively; kanamycin (50 µg/ml), ampicillin (100 µg/ml), and tetracycline (20 µg/ml) for *E.coli* strain.

### Cell culture

Both the Lepidoptera insect *Spodoptera frugiperda* Sf9 cell line and *Lymantria dispar* IPLB LD-652Y-5d cell line were provided by Dr. C.H. Wang (Department of Entomology, National Taiwan University). The gypsy moth (*Lymantria dispar*) cell line, IPLB LD-652Y-5d was subcloned from IPLB LD-652Y [53]. They were grown in Sf-900 II SFM (Gibco) medium supplemented with 10% fetal bovine serum (FBS) and 1% penicillin/streptomycin/glutamine (PSG) (Invitrogen) at 27°C.

### Construction of the *P. taiwanensis* Δ*tccC* knockout mutant

An *tccC* (GenBank database accession number, HQ260745) knockout mutant of *P. taiwanensis*, designated Δ*tccC* was constructed by double recombination of the suicide vector pEX100T [54] containing the *tccC* fragment with a kanamycin resistance cassette inserted [55]. A *tccC-kan-tccC* fragment was generated by inserting a 1345-bp kanamycin resistance cassette into an 852-bp fragment that contains the coding sequence of *tccC*. The *tccC-kan-tccC* fragment was cloned into pEX100T suicide vector, and then transformed into *E.coli* S17-1 for conjugation with wild-type *P. taiwanensis*. The double recombination *tccC* mutant strain was selected on LB plates containing 5% sucrose, 30 µg/ml kanamycin, 34 µg/ml rifampicin, and 100 µg/ml spectinomycin. The resulting Δ*tccC* mutant was confirmed by PCR and sequencing.

### Bioassay of infection experiments and effective protein fractions

Bioassays of bacteria infection of larvae were performed by natural oral infection. *P. taiwanensis* was grown for 24 hours to the stationary phase and collected. Subsequently, the cell pellet was washed three times in 5 ml PBS (pH 7.4) and resuspended in PBS, adjusted to different concentrations (OD). Different concentrations of bacteria (50 µl) were applied to surface of 0.5×1 cm^2^ vegetable pieces, which were used for feeding larvae of vegetable moth *Plutella xylostella* and incubated at 25°C. Each infected larva was observed at day 5 after oral infection and the mortality rate was calculated. Healthy third-instar *P. xylostella* larvae were provided by the Taiwan Agricultural Chemicals and Toxic Substances Research Institute.

To determine the protein fractions that cause mortality against *P. xylostella*, *P. taiwanensis* was cultured for 24 hours. The cell culture was harvested by centrifugation (15 min at 4,600*g*, 4°C), and supernatants and cell pellets were collected separately. For culture supernatants, the secreted proteins were filtered through a 0.22 µm PVDF filter (Millipore) and concentrated using a Vivaspin 20 concentrator (10 kDa MWCO, GE Healthcare). The harvested cell pellets were washed with PBS two times and resuspended in PBS with protease inhibitor and lysed with sonication (cell lysates). The cell lystaes were separated into insoluble lysates and soluble lysates by centrifugation (30 min at 26,000*g*, 4°C), and the soluble lysates were filtered by a 0.22 µm PVDF filter. The insoluble lysates were washed with PBS two times and resuspended in PBS. A schematic of the experimental procedure for extraction of the various protein fractions of *P. taiwanensis* is shown in Figure S3. For toxicity analysis of protein fractions from *P. taiwanensis*, 300 ng of proteins dissolved in 10 µl PBS were used for insect larvae treatment. Protein extracts were quantified by Pierce 660 nm protein assay method (Pierce).

### Cell survival assay

To investigate the effect of *P. taiwanensis* on insect cells, proliferation of *Spodoptera frugiperda* Sf9 cells was determined by a colorimetric XTT assay [56]. For cytotoxicity assay, Sf9 cells were seeded at 5,000 per well in 96-well culture plates supplemented with 10 µg/ml of the various fraction proteins of *P. taiwanensis* (extraction procedure shown in Figure S3), or a multiplicity of infection (MOI) of 1000 *Pt/cell* was added in antibiotic-free medium. After 72-h treatment, cell proliferation was quantified by Cell Proliferation Assay Kit (XTT) (Biological Industries).

### Apoptotic assay

Cell early stage apoptosis was detected by Annexin V-FITC assay [57]. The percentages of apoptosis of human or insect cells were determined by counting visible annexin V-positive cells under the fluorescence microscope. Cells (5,000 cells well^−1^) were incubated with protein fractions of *P. taiwanensis* at 10 µg/ml or with *P. taiwanensis* (MOI = 1000) for 72 h on the well in 24-well plates. After treatment for 72 h, the cells were washed twice in PBS and detected using the ApoAlert Annexin V-FITC Kit (BD) according to the manufacturer's instructions. The DNA in the nuclei was stained with 4′,6-diamidino-2-phenylindole dilactate (DAPI) for 5 min. Finally, the stained cells were washed twice in PBS, fixed with 4% paraformaldehyde for 10 minutes, and then observed under a fluorescence microscope (Zeiss Axiovert 100M, Carl Zeiss, Germany). Annexin V positive cells were counted and identified as *P. taiwanensis*-induced early stage apoptotic cells.

### Sectioning and HE, gram, immunohistochemistry staining

After bacteria oral infection for 48 h, third instar larvae were fixed in 10% buffered formalin (pH 7.0) for at least 48 h. After fixation, larvae were sent to the Laboratory of Pathological Section of National Taiwan University for sectioning. The tissue sections were analyzed by hematoxylin–eosin, Gram's, or immunohistochemistry staining. Immunohistochemical (IHC) staining was performed using anti-JNK-2 [N1C3] (GTX105523, Genetex; 80% [276/398] sequence identity to c-Jun NH2-terminal kinase of *Bombyx mori*, NP_001103396) and anti caspase-3 p17 (GTX123678, Genetex; 36% [46/129] sequence identity to caspase 3 of *Bombyx mori*, AAW79564 [58]) antibodies, followed by diaminobenzidine (DAB) for color development and counterstained with hematoxylin from the Laboratory Animal Center of National Taiwan University Hospital.

### Purification of TccC

Full-length TccC-His_6_ fusion fragment was cloned into the broad host range Pcpp30 vector and transformed into *E. coli* (BL21) and *P. taiwanensis*. Overexpressed TccC-His_6_ fusion protein was purified by His SpinTrap columns (GE Healthcare) after *P. taiwanensis* and *E. coli* growth into stationary phase (24 h), and the results were displayed by western blotting using the anti-TccC antibody.

### Analysis of TccC location

#### Western immunoblotting

For SDS PAGE, 20 µg proteins of different cellular fractions from *P. taiwanensis* were dissolved in loading buffer with SDS and then applied to gel electrophoresis. After electrophoresis, the proteins were transferred to nitrocellulose membranes under 40 mA for 12 h. TccC was detected with specific anti-TccC antibody, using rabbit polyclonal antibodies raised against *P. taiwanensis* TccC full-length recombinant protein purified from *E.coli* BL21 expression. After first antibody binding, the color was developed with horseradish peroxidase-coupled anti-rabbit secondary antibody binding and chemiluminescent detection reagent (Pierce).

#### Flow cytometry

Flow cytometry was used to determine membrane localization of TccC. Wild-type and ΔTccC mutant strains of *P. taiwanensis* were grown overnight and collected at stationary phase (24 h). The cultures were adjusted 10^9^ CFU/ml, and then 100 µl adjusted-bacteria was centrifuged to collect pellets. The bacteria pellets were washed three times with PBS at 4°C and resuspended in 200 µl PBS with 1% BSA. The polyclonal anti-TccC antibody (1/100 dilution) was added to the bacteria suspension on ice for 1 h. The bacteria was washed three times with PBS again and stained with goat FITC-conjugated anti-rabbit IgG secondary antibody (1/100 dilution) (Jackson Immunoresearch) on ice for 1 h. After staining, the bacteria were washed three times and resuspended in 1 ml PBS and analyzed by flow cytometry. Flow cytometry was performed by MoFlo XDP Cell Sorter (Beckman Coulter) using Summit 5.2 software (Beckman Coulter).

### Phagocytosis assay

*P. taiwanensis* cells were collected in the early stationary phase and washed twice with PBS, and resuspended in PBS to OD = 1 (4×10^9^ cells). One milliliter of resuspended cells was added to CFSE (final concentration of 5 µM) and incubated at 30°C in the dark for 30 min. The cells were washed three times with PBS and observed under fluorescent microscope. For phagocytosis assays, CSFE labeled *P. taiwanensis* cells were added to macrophage cells (MOI = 1000) for 30 min at 37°C in the dark, and then washed three times with PBS. Quantification and observation of phagocytosis was measured by flow cytometry and fluorescent microscope respectively. Flow cytometry was performed by Cytomics FC500 (Beckman Coulter) using CXP software (Beckman Coulter). Ten thousand cells were collected for analyses. Non-infected macrophage cells were used as a negative control.

### Quantitative H_2_O_2_ assay and proliferation assay of *P. taiwanensis*

*P. taiwanensis* cells grown to stationary phase (24 h) were collected, washed three times in PBS, and resuspended in PBS to 10^9^ cells per ml and subsequently incubated with 1M H_2_O_2_. The concentration of H_2_O_2_ remaining was detected at different time points after treatments using a PeroX-Oquant Quantitative Peroxide Assay Kits (Pierce).

Visualization of the proliferation effect of hydroxyl radicals in *P. taiwanensis* was performed as described in Huang and Chiou (2011) [59]. *P. taiwanensis* was grown in LB broth for 24 h and then incubated with different concentrations of H_2_O_2_ for 3 h. Proliferation was determined by counting the colony-forming units.

### Quantitative ^15^N-L-glutamate uptake assay in *P. taiwanensis*

*P. taiwanensis* cells grown to the stationary phase (24 h) were collected, and cultured with LB broth containing 250 µM of ^15^N-L-glutamate for additional 4 hours. The cells were washed three times with PBS and dried at 80°C for 2 days. The dried cells were weighed and the^15^N content was analyzed using a continuous-flow isotope ratio mass spectrometer coupled with a carbon nitrogen elemental analyzer (ANCA-GSL 20/20; PDZ Europa).

## Supporting Information

Figure S1 **Diagram of TccC-like proteins in different insect pathogens.** The N-terminus of the *tccC*-like gene encodes the highly conserved region of RhsA protein within the Rhs repeat-associated core domain and the C-terminus displays a hypervariable region among different entomopathogenic bacteria. The C-terminus of the TccC region in *P. taiwanensis* displays amino acid similarity with the sodium/glutamate symporter and the TraT complement resistance proteins. So far, only *P. luminescens* TccC5 has shown ADP-ribosyltransferase function [5], whereas the functions of other TccCs are unclear. The proteins and the domains were predicted by the NCBI Conserved Domain Database (CDD) and the Pfam Protein Families database. The deduced amino acid sequences of TccC were obtained from the NCBI databases as follows: *Pseudomonas taiwanensis* TccC1 (accession no. ADO85706), TccC2 (unpublished whole genome database); *Pseudomonas entomophila* (accession no. CAK15567); *Xenorhabdus bovienii* TccC (accession no. YP_003467480); *Xenorhabdus nematophila* TccC1 (accession no. YP_003712427), TccC2 (accession no. YP_003712779); *Photorhabdus luminescens* TccC1 (accession no. NP_931350), TccC2 (accession no. AAL18492), TccC3 (accession no. AAO17204), TccC5 (accession no. AAO17210); *Photorhabdus asymbiotica* TccC2 (accession no. CAQ82972); *Bacillus thuringiensis* (accession no. EEM92890).
(DOCX)

Figure S2 **Schematic representation of insertion of** ***kannmycin*** **cassette and RT-PCR analysis of** ***tccC*** **gene expression in wild-type and** ***tccC*** **mutant strains.** (A) Schematic illustration of insertion of kanamycin upstream of the RhsA-like domain of the *tccC* gene. (B) The kan insertion region was checked by PCR. (C) *tccC* expression of *tccC* mutant strains were confirmed by RT-PCR.
(DOCX)

Figure S3 **Schematic of experimental procedure for separating different protein fractions from** ***P. taiwanensis*** **.**
(DOCX)

Figure S4 **Interaction of Sf9 insect cells and RFP-labeled** ***P. taiwanensis*** **.** (A) RFP-labelled *P. taiwanensis* was incubated for 1 h with Sf9 cells and observed by confocal microscope. (B) Non-infection of control Sf9 cells. (C) Lysis of Sf9 cells was observed by light microscope after interaction for 3 h. Scale bar = 5 µm (left panel); 10 µm (right panel).
(DOCX)

Figure S5 **Determination of protease activity in** ***P. taiwanensis*** **.** Proteolytic activity of the culture supernatant of *P. taiwanensis* in early (24 h), middle (36 h), and late (48 h) stationary phase was measured by azocasein substrate at 440 nm.
(DOCX)

Figure S6 **Comparison of hydrogen peroxide decomposition by wild-type and** ***tccC*** **mutant of** ***P. taiwanensis*** **.** Degradation of hydrogen peroxide was measured in the culture medium at different time points.
(DOCX)

Figure S7 **TccC promotes glutamate uptake activity in** ***P. taiwanensis*** **.** Wild-type and ΔtccC mutant *P. taiwanensis* cells were cultured in medium containing 250 µM of ^15^N-L-glutamate for 4 hours. Uptake activity of ^15^N-L-glutamate was defined as enrichment of ^15^N content in *P. taiwanensis* cells. The ^15^N enrichment was calculated as ^15^N abundance in labeled cells minus ^15^N abundance in non-labeled cells. The asterisk indicates a P value = 0.035 from triplet repeats, as determined by Student's *t*-test.
(DOCX)

Figure S8 **Survival of macrophages after treatment with wild-type or** ***tccC*** **mutant of** ***P. taiwanensis*** ***in vitro.*** Macrophages incubated with wild-type or tccC mutant of *P. taiwanensis* (MOI = 1000) for 24 h and survival rates were detected by XTT assay.
(DOCX)

Figure S9 **Western blot analysis of cleavage of the C-terminal TccC fragment by immobilized metal affinity column.** Overexpression of the whole length *tccC* gene in *E. coli* and *P. taiwanensis* by broad host vector pCPP30. Cleaved TccC was purified by immobilized metal affinity column and detected by TccC antibody.
(DOCX)

Table S1 **Rough screening tests of mortality of insect larvae after oral infection with** ***P. taiwanensis*** **.** *P. taiwanensis* was grown overnight and collected. Each bacteria fermentation (30 µl) was individually applied to 10 surfaces of 0.5×1 cm^2^ vegetable pieces for 30 larvae, which were used for feeding larvae and incubated at 25°C. Each infected larva was observed at day 5 after oral infection and the mortality rate was calculated. The two-tail student t-test was used to determine statistical significance. Each treatment (30 larvae) was repeated three times. The control was germ-free LB medium treatment.
(DOCX)

## References

[1] Waterfield NR, Bowen DJ, Fetherston JD, Perry RD, ffrench-Constant RH (2001) The tc genes of *Photorhabdus*: a growing family. Trends Microbiol 9: 185–191. 11286884 10.1016/s0966-842x(01)01978-3

[2] Ffrench-Constant R, Waterfield N (2006) An ABC guide to the bacterial toxin complexes. Adv Appl Microbiol 58: 169–183. 16509446

[3] Waterfield N, Dowling A, Sharma S, Daborn PJ, Potter U, et al (2001) Oral toxicity of *Photorhabdus luminescens* W14 toxin complexes in *Escherichia coli* . Appl Environ Microbiol 67: 5017–5024. 11679320 10.1128/AEM.67.11.5017-5024.2001 PMC93265

[4] Waterfield N, Hares M, Yang G, Dowling A, ffrench-Constant R (2005) Potentiation and cellular phenotypes of the insecticidal Toxin complexes of *Photorhabdus* bacteria. Cellular Microbiology 7: 373–382. 15679840 10.1111/j.1462-5822.2004.00467.x

[5] Lang AE, Schmidt G, Schlosser A, Hey TD, Larrinua IM, et al (2010) *Photorhabdus luminescens* Toxins ADP-Ribosylate Actin and RhoA to Force Actin Clustering. Science 327: 1139–1142. 20185726 10.1126/science.1184557

[6] Liu D, Burton S, Glancy T, Li Z-S, Hampton R, et al (2003) Insect resistance conferred by 283-kDa *Photorhabdus luminescens* protein TcdA in Arabidopsis thaliana. Nature Biotechnology 21: 1222–1228. 10.1038/nbt866 12949536

[7] Lee SC, Stoilova-Mcphie S, Baxter L, Fülöp V, Henderson J, et al (2007) Structural Characterisation of the Insecticidal Toxin XptA1, Reveals a 1.15 MDa Tetramer with a Cage-like Structure. Journal of Molecular Biology 366: 1558–1568. 17266984 10.1016/j.jmb.2006.12.057

[8] Otto H, Tezcan-Merdol D, Girisch R, Haag F, Rhen M, et al (2000) The spvB gene-product of the *Salmonella enterica* virulence plasmid is a mono(ADP-ribosyl)transferase. Mol Microbiol 37: 1106–1115. 10972829 10.1046/j.1365-2958.2000.02064.x

[9] Moore JD (2001) The Ran-GTPase and cell-cycle control. Bioessays 23: 77–85. 11135312 10.1002/1521-1878(200101)23:1<77::AID-BIES1010>3.0.CO;2-E

[10] Joo Lee P, Ahn J-Y, Kim Y-H, Wook Kim S, Kim J-Y, et al (2004) Cloning and heterologous expression of a novel insecticidal gene (tccC1) from *Xenorhabdus nematophilus* strain. Biochemical and Biophysical Research Communications 319: 1110–1116. 15194482 10.1016/j.bbrc.2004.04.203

[11] Lemaitre B, Hoffmann J (2007) The Host Defense of *Drosophila melanogaster* . Annual Review of Immunology 25: 697–743. 10.1146/annurev.immunol.25.022106.141615 17201680

[12] Ha EM, Oh CT, Bae YS, Lee WJ (2005) A direct role for dual oxidase in *Drosophila* gut immunity. Science 310: 847–850. 16272120 10.1126/science.1117311

[13] Ryu JH, Ha EM, Oh CT, Seol JH, Brey PT, et al (2006) An essential complementary role of NF-kappaB pathway to microbicidal oxidants in *Drosophila* gut immunity. EMBO J 25: 3693–3701. 16858400 10.1038/sj.emboj.7601233 PMC1538556

[14] Liehl P, Blight M, Vodovar N, Boccard F, Lemaitre B (2006) Prevalence of Local Immune Response against Oral Infection in a *Drosophila*/*Pseudomonas* Infection Model. PLoS Pathogens 2: e56. 16789834 10.1371/journal.ppat.0020056 PMC1475658

[15] Vodovar N, Vallenet D, Cruveiller S, Rouy Z, Barbe V, et al (2006) Complete genome sequence of the entomopathogenic and metabolically versatile soil bacterium *Pseudomonas entomophila* . Nature Biotechnology 24: 673–679. 10.1038/nbt1212 16699499

[16] Vodovar N (2005) Drosophila host defense after oral infection by an entomopathogenic *Pseudomonas* species. Proceedings of the National Academy of Sciences 102: 11414–11419. 10.1073/pnas.0502240102 PMC1183552 16061818

[17] Buchon N, Broderick NA, Chakrabarti S, Lemaitre B (2009) Invasive and indigenous microbiota impact intestinal stem cell activity through multiple pathways in *Drosophila* . Genes Dev 23: 2333–2344. 19797770 10.1101/gad.1827009 PMC2758745

[18] Apidianakis Y, Pitsouli C, Perrimon N, Rahme L (2009) Synergy between bacterial infection and genetic predisposition in intestinal dysplasia. Proc Natl Acad Sci U S A 106: 20883–20888. 19934041 10.1073/pnas.0911797106 PMC2791635

[19] Jiang H, Patel PH, Kohlmaier A, Grenley MO, McEwen DG, et al (2009) Cytokine/Jak/Stat signaling mediates regeneration and homeostasis in the Drosophila midgut. Cell 137: 1343–1355. 19563763 10.1016/j.cell.2009.05.014 PMC2753793

[20] Wang LT, Tai CJ, Wu YC, Chen YB, Lee FL, et al (2009) *Pseudomonas taiwanensis* sp. nov., isolated from soil. International Journal of Systematic and Evolutionary Microbiology 60: 2094–2098. 19854877 10.1099/ijs.0.014779-0

[21] Wang S-L, Chen S-J, Wang C-L (2008) Purification and characterization of chitinases and chitosanases from a new species strain *Pseudomonas* sp. TKU015 using shrimp shells as a substrate. Carbohydrate Research 343: 1171–1179. 18378219 10.1016/j.carres.2008.03.018

[22] Wang S-L, Chen H-J, Liang T-W, Lin Y-D (2009) A novel nattokinase produced by *Pseudomonas* sp. TKU015 using shrimp shells as substrate. Process Biochemistry 44: 70–76.

[23] Liu J-R, Lin Y-D, Chang S-T, Zeng Y-F, Wang S-L (2010) Molecular Cloning and Characterization of an Insecticidal Toxin from *Pseudomonas taiwanensis* . Journal of Agricultural and Food Chemistry 58: 12343–12349. 21062004 10.1021/jf103604r

[24] Lang AE, Schmidt G, Sheets JJ, Aktories K (2011) Targeting of the actin cytoskeleton by insecticidal toxins from *Photorhabdus luminescens* . Naunyn Schmiedebergs Arch Pharmacol 383: 227–235. 21072628 10.1007/s00210-010-0579-5

[25] Hakim RS, Baldwin K, Smagghe G (2010) Regulation of Midgut Growth, Development, and Metamorphosis. Annual Review of Entomology 55: 593–608. 10.1146/annurev-ento-112408-085450 19775239

[26] Hurst MRH, Jones SA, Binglin T, Harper LA, Jackson TA, et al (2011) The Main Virulence Determinant of *Yersinia entomophaga* MH96 Is a Broad-Host-Range Toxin Complex Active against Insects. Journal of Bacteriology 193: 1966–1980. 21278295 10.1128/JB.01044-10 PMC3133040

[27] Loeb MJ, Martin PA, Hakim RS, Goto S, Takeda M (2001) Regeneration of cultured midgut cells after exposure to sublethal doses of toxin from two strains of *Bacillus thuringiensis* . J Insect Physiol 47: 599–606. 11249948 10.1016/s0022-1910(00)00150-5

[28] Tanaka S, Yoshizawa Y, Sato R (2012) Response of midgut epithelial cells to Cry1Aa is toxin-dependent and depends on the interplay between toxic action and the host apoptotic response. FEBS Journal 279: 1071–1079. 22260394 10.1111/j.1742-4658.2012.08499.x

[29] Meneses-Acosta A, Mendonca R, Merchant H, Covarrubias L, Ramirez O (2001) Comparative characterization of cell death between Sf9 insect cells and hybridoma cultures. Biotechnol Bioeng 72: 441–457. 11180064

[30] Ryoo HD, Gorenc T, Steller H (2004) Apoptotic cells can induce compensatory cell proliferation through the JNK and the Wingless signaling pathways. Dev Cell 7: 491–501. 15469838 10.1016/j.devcel.2004.08.019

[31] Shahidi-Noghabi S, Damme EJMV, Iga M, Smagghe G (2010) Exposure of insect midgut cells to *Sambucus nigra* L. agglutinins I and II causes cell death via caspase-dependent apoptosis. Journal of Insect Physiology 56: 1101–1107. 20230823 10.1016/j.jinsphys.2010.03.012

[32] Bradshaw M, Dineen SS, Maks ND, Johnson EA (2004) Regulation of neurotoxin complex expression in *Clostridium botulinum* strains 62A, Hall A-hyper, and NCTC 2916. Anaerobe 10: 321–333. 16701534 10.1016/j.anaerobe.2004.07.001

[33] Saujet L, Monot M, Dupuy B, Soutourina O, Martin-Verstraete I (2011) The key sigma factor of transition phase, SigH, controls sporulation, metabolism, and virulence factor expression in *Clostridium difficile* . Journal of bacteriology 193: 3186–3196. 21572003 10.1128/JB.00272-11 PMC3133256

[34] Whistler CA, Corbell NA, Sarniguet A, Ream W, Loper JE (1998) The two-component regulators GacS and GacA influence accumulation of the stationary-phase sigma factor sigmaS and the stress response in *Pseudomonas fluorescens* Pf-5. J Bacteriol 180: 6635–6641. 9852008 10.1128/jb.180.24.6635-6641.1998 PMC107767

[35] De la Torre-Zavala S, Aguilera S, Ibarra-Laclette E, Hernandez-Flores JL, Hernandez-Morales A, et al (2011) Gene expression of Pht cluster genes and a putative non-ribosomal peptide synthetase required for phaseolotoxin production is regulated by GacS/GacA in *Pseudomonas syringae* pv. *phaseolicola* . Res Microbiol 162: 488–498. 21527339 10.1016/j.resmic.2011.04.010

[36] Ha EM, Oh CT, Ryu JH, Bae YS, Kang SW, et al (2005) An antioxidant system required for host protection against gut infection in *Drosophila* . Developmental cell 8: 125–132. 15621536 10.1016/j.devcel.2004.11.007

[37] Tzou P, De Gregorio E, Lemaitre B (2002) How *Drosophila* combats microbial infection: a model to study innate immunity and host-pathogen interactions. Current opinion in microbiology 5: 102–110. 11834378 10.1016/s1369-5274(02)00294-1

[38] Hultmark D (2003) *Drosophila* immunity: paths and patterns. Current opinion in immunology 15: 12–19. 12495727 10.1016/s0952-7915(02)00005-5

[39] Meusch D, Gatsogiannis C, Efremov RG, Lang AE, Hofnagel O, et al (2014) Mechanism of Tc toxin action revealed in molecular detail. Nature 508: 61–65. 24572368 10.1038/nature13015

[40] Kalman M, Gentry DR, Cashel M (1991) Characterization of the *Escherichia coli* K12 *gltS* glutamate permease gene. Mol Gen Genet 225: 379–386. 2017136 10.1007/BF00261677

[41] Tolner B, Poolman B, Konings WN (1992) Characterization and functional expression in *Escherichia coli* of the sodium/proton/glutamate symport proteins of *Bacillus stearothermophilus* and *Bacillus caldotenax* . Mol Microbiol 6: 2845–2856. 1359385 10.1111/j.1365-2958.1992.tb01464.x

[42] Shibayama K, Wachino J, Arakawa Y, Saidijam M, Rutherford NG, et al (2007) Metabolism of glutamine and glutathione via gamma-glutamyltranspeptidase and glutamate transport in Helicobacter pylori: possible significance in the pathophysiology of the organism. Mol Microbiol 64: 396–406. 17381553 10.1111/j.1365-2958.2007.05661.x

[43] Hendriksen WT, Kloosterman TG, Bootsma HJ, Estevao S, de Groot R, et al (2008) Site-specific contributions of glutamine-dependent regulator GlnR and GlnR-regulated genes to virulence of *Streptococcus pneumoniae* . Infect Immun 76: 1230–1238. 18174343 10.1128/IAI.01004-07 PMC2258823

[44] Tala A, Monaco C, Nagorska K, Exley RM, Corbett A, et al (2011) Glutamate utilization promotes meningococcal survival in vivo through avoidance of the neutrophil oxidative burst. Molecular microbiology 81: 1330–1342. 21777301 10.1111/j.1365-2958.2011.07766.x PMC3755445

[45] Donaldson DM, Roberts RR, Larsen HS, Tew JG (1974) Interrelationship between serum beta-lysin, lysozyme, and the antibody-complement system in killing *Escherichia coli* . Infect Immun 10: 657–666. 4609906 10.1128/iai.10.3.657-666.1974 PMC423000

[46] Patterson-Delafield J, Martinez RJ, Lehrer RI (1980) Microbicidal cationic proteins in rabbit alveolar macrophages: a potential host defense mechanism. Infect Immun 30: 180–192. 7439972 10.1128/iai.30.1.180-192.1980 PMC551293

[47] Aguero ME, Aron L, DeLuca AG, Timmis KN, Cabello FC (1984) A plasmid-encoded outer membrane protein, TraT, enhances resistance of *Escherichia coli* to phagocytosis. Infect Immun 46: 740–746. 6389361 10.1128/iai.46.3.740-746.1984 PMC261607

[48] Au C, Dean P, Reynolds SE, ffrench-Constant RH (2004) Effect of the insect pathogenic bacterium *Photorhabdus* on insect phagocytes. Cell Microbiol 6: 89–95. 14678333 10.1046/j.1462-5822.2003.00345.x

[49] Sukupolvi S, O'Connor CD (1990) TraT lipoprotein, a plasmid-specified mediator of interactions between gram-negative bacteria and their environment. Microbiol Rev 54: 331–341. 2087219 10.1128/mr.54.4.331-341.1990 PMC372785

[50] Tomb JF, White O, Kerlavage AR, Clayton RA, Sutton GG, et al (1997) The complete genome sequence of the gastric pathogen *Helicobacter pylori* . Nature 388: 539–547. 9252185 10.1038/41483

[51] Fan Y, Bergmann A (2010) The cleaved-Caspase-3 antibody is a marker of Caspase-9-like DRONC activity in *Drosophila* . Cell Death Differ 17: 534–539. 19960024 10.1038/cdd.2009.185 PMC2822068

[52] Simon R, Priefer U, Puhler A (1983) A Broad Host Range Mobilization System for In Vivo Genetic Engineering: Transposon Mutagenesis in Gram Negative Bacteria. Nat Biotech 1: 784–791.

[53] McClintock JT, Dougherty EM, Weiner RM (1986) Semipermissive Replication of a Nuclear Polyhedrosis Virus of Autographa californica in a Gypsy Moth Cell Line. J Virol 57: 197–204. 16789253 10.1128/jvi.57.1.197-204.1986 PMC252715

[54] Schweizer HP, Hoang TT (1995) An improved system for gene replacement and xylE fusion analysis in *Pseudomonas aeruginosa* . Gene 158: 15–22. 7789804 10.1016/0378-1119(95)00055-b

[55] Datsenko KA (2000) One-step inactivation of chromosomal genes in *Escherichia coli* K-12 using PCR products. Proceedings of the National Academy of Sciences 97: 6640–6645. 10.1073/pnas.120163297 PMC18686 10829079

[56] Roehm NW, Rodgers GH, Hatfield SM, Glasebrook AL (1991) An improved colorimetric assay for cell proliferation and viability utilizing the tetrazolium salt XTT. J Immunol Methods 142: 257–265. 1919029 10.1016/0022-1759(91)90114-u

[57] van Engeland M, Nieland LJ, Ramaekers FC, Schutte B, Reutelingsperger CP (1998) Annexin V-affinity assay: a review on an apoptosis detection system based on phosphatidylserine exposure. Cytometry 31: 1–9. 9450519 10.1002/(sici)1097-0320(19980101)31:1<1::aid-cyto1>3.0.co;2-r

[58] Porta H, Muñoz-Minutti C, Soberón M, Bravo A (2011) Induction of Manduca sexta Larvae Caspases Expression in Midgut Cells by *Bacillus thuringiensis* Cry1Ab Toxin. Psyche: A Journal of Entomology 2011: 1–7.

[59] Huang C-H, Chiou S-H (2011) Proteomic analysis of upregulated proteins in *Helicobacter pylori* under oxidative stress induced by hydrogen peroxide. The Kaohsiung Journal of Medical Sciences 27: 544–53. 22208537 10.1016/j.kjms.2011.06.019 PMC11916125

