# Supplementary material for: Characterization of an Insecticidal Toxin and Pathogenicity of Pseudomonas taiwanensis against Insects
Source: PLoS Pathog. 2014 Aug 21;10(8):e1004288. doi: 10.1371/journal.ppat.1004288 (PMC4140846; doi:10.1371/journal.ppat.1004288)
Supplement: Figure S1 — Diagram of TccC-like proteins in different insect pathogens. The N-terminus of the tccC-like gene encodes the highly conserved region of RhsA protein within the Rhs repeat-associated core domain and the C-terminus displays a hypervariable region among different entomopathogenic bacteria. The C-terminus of the TccC region in P. taiwanensis displays amino acid similarity with the sodium/glutamate symporter and the TraT complement resistance proteins. So far, only P. luminescens TccC5 has shown ADP-ribosyltransferase function [5], whereas the functions of other TccCs are unclear. The proteins and the domains were predicted by the NCBI Conserved Domain Database (CDD) and the Pfam Protein Families database. The deduced amino acid sequences of TccC were obtained from the NCBI databases as follows: Pseudomonas taiwanensis TccC1 (accession no. ADO85706), TccC2 (unpublished whole genome database); Pseudomonas entomophila (accession no. CAK15567); Xenorhabdus bovienii TccC (accession no. YP_003467480); Xenorhabdus nematophila TccC1 (accession no. YP_003712427), TccC2 (accession no. YP_003712779); Photorhabdus luminescens TccC1 (accession no. NP_931350), TccC2 (accession no. AAL18492), TccC3 (accession no. AAO17204), TccC5 (accession no. AAO17210); Photorhabdus asymbiotica TccC2 (accession no. CAQ82972); Bacillus thuringiensis (accession no. EEM92890). (DOCX) [file ppat.1004288.s001.docx]

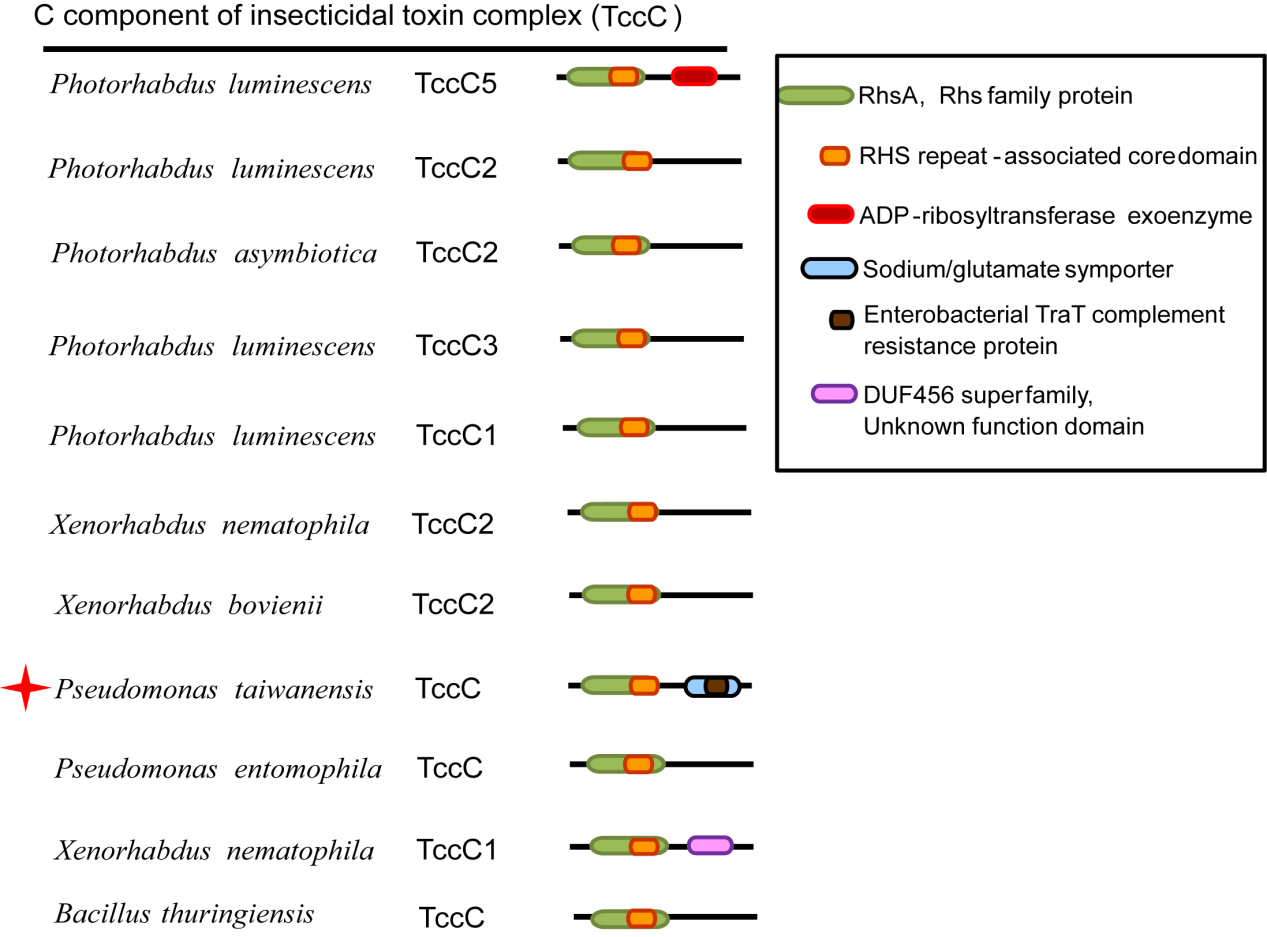


**Figure S1.** **Diagram of TccC-like proteins in different insect pathogens.** The N-terminus of the *tccC*-like gene encodes the highly conserved region of RhsA protein within the Rhs repeat-associated core domain and the C-terminus displays a hypervaraible region among different entomopathogenic bacteria. The C-terminus of the TccC region in *P. taiwanensis* displays amino acid similarity with the sodium/glutamate symporter and the TraT complement resistance proteins. So far, only *P. luminescens* TccC5 has shown ADP-ribosyltransferase function [5], whereas the functions of other TccCs are unclear. The proteins and the domains were predicted by the NCBI Conserved Domain Database (CDD) and the Pfam Protein Families database. The deduced amino acid sequences of TccC were obtained from the NCBI databases as follows: *Pseudomonas taiwanensis* TccC1 (accession no. ADO85706), TccC2 (unpublished whole genome database); *Pseudomonas entomophila* (accession no. CAK15567); *Xenorhabdus bovienii* TccC (accession no. YP_003467480*)*; *Xenorhabdus nematophila* TccC1 (accession no. YP_003712427), TccC2 (accession no. YP_003712779); *Photorhabdus luminescens* TccC1 (accession no. NP_931350), TccC2 (accession no. AAL18492), TccC3 (accession no. AAO17204), TccC5 (accession no. AAO17210); *Photorhabdus asymbiotica* TccC2 (accession no. CAQ82972); *Bacillus thuringiensis* (accession no. EEM92890).
