# Supplementary material for: Characterization of an Insecticidal Toxin and Pathogenicity of Pseudomonas taiwanensis against Insects
Source: PLoS Pathog. 2014 Aug 21;10(8):e1004288. doi: 10.1371/journal.ppat.1004288 (PMC4140846; doi:10.1371/journal.ppat.1004288)
Supplement: Figure S2 — Schematic representation of insertion of kannmycin cassette and RT-PCR analysis of tccC gene expression in wild-type and tccC mutant strains. (A) Schematic illustration of insertion of kanamycin upstream of the RhsA-like domain of the tccC gene. (B) The kan insertion region was checked by PCR. (C) tccC expression of tccC mutant strains were confirmed by RT-PCR. (DOCX) [file ppat.1004288.s002.docx]

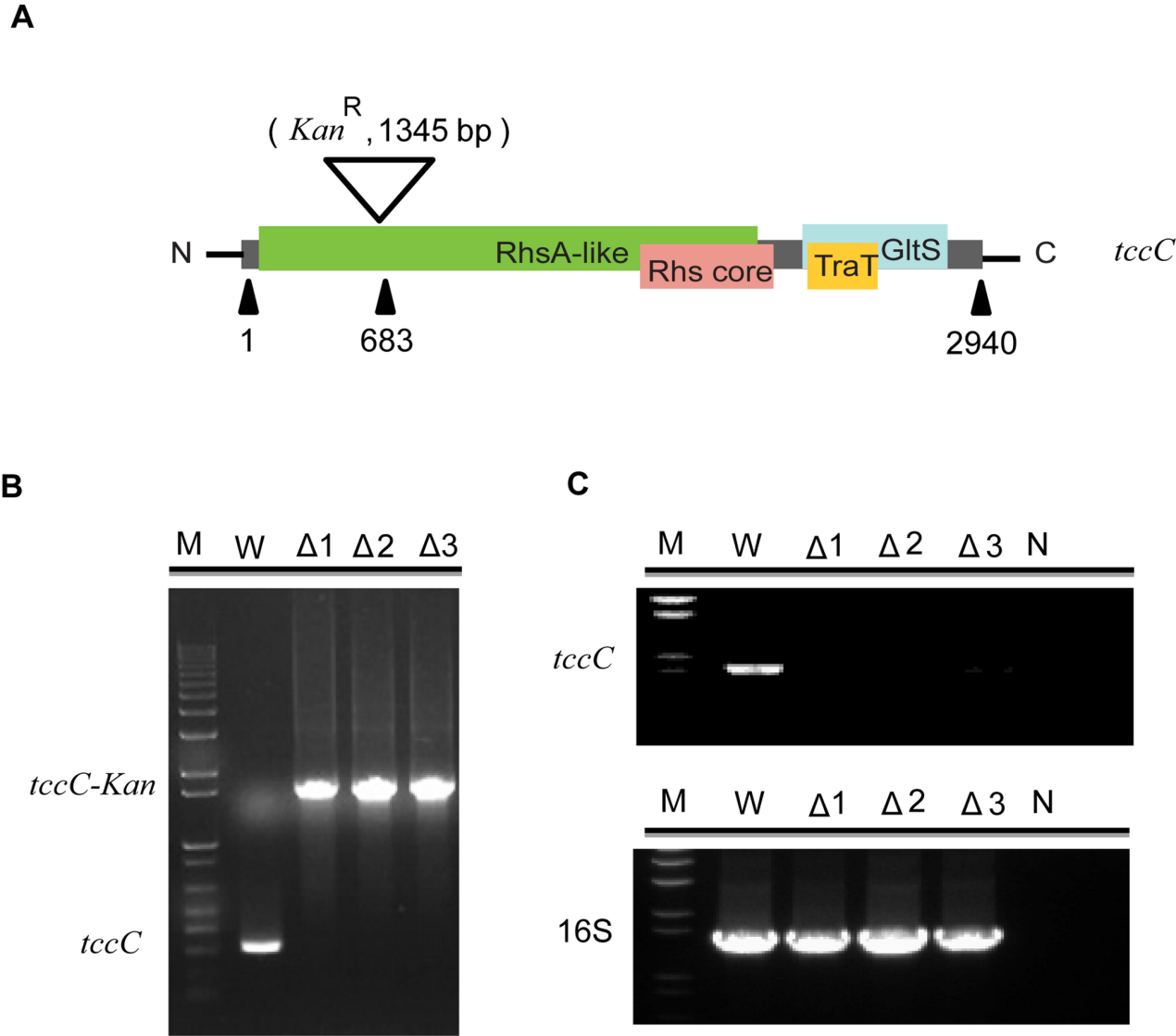


**Figure S2.** **Schematic representation of insertion of *kannmycin* cassette and RT-PCR analysis of *tccC* gene expression in wild-type and *tccC* mutant strains**. (A) Schematic illustration of insertion of kanamycin upstream of the RhsA-like domain of the *tccC* gene. (B) The kan insertion region was checked by PCR. (C) *tccC* expression of *tccC* mutant strains were confirmed by RT-PCR.
