# Supplementary material for: Characterization of an Insecticidal Toxin and Pathogenicity of Pseudomonas taiwanensis against Insects
Source: PLoS Pathog. 2014 Aug 21;10(8):e1004288. doi: 10.1371/journal.ppat.1004288 (PMC4140846; doi:10.1371/journal.ppat.1004288)
Supplement: Figure S3 — Schematic of experimental procedure for separating different protein fractions from P. taiwanensis . (DOCX) [file ppat.1004288.s003.docx]

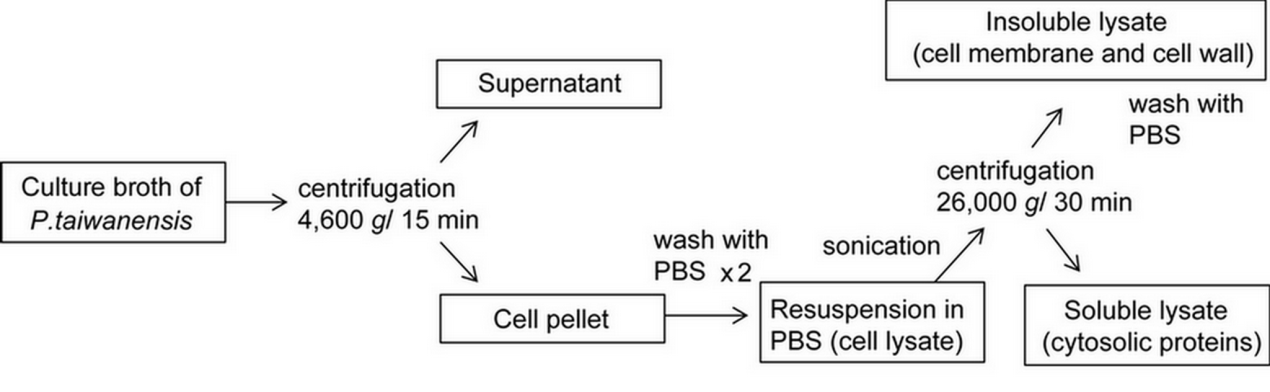


**Figure S3. Schematic of experimental procedure for separating different protein fractions from *P. taiwanensis*.**
