# Supplementary material for: Characterization of an Insecticidal Toxin and Pathogenicity of Pseudomonas taiwanensis against Insects
Source: PLoS Pathog. 2014 Aug 21;10(8):e1004288. doi: 10.1371/journal.ppat.1004288 (PMC4140846; doi:10.1371/journal.ppat.1004288)
Supplement: Figure S4 — Interaction of Sf9 insect cells and RFP-labeled P. taiwanensis . (A) RFP-labelled P. taiwanensis was incubated for 1 h with Sf9 cells and observed by confocal microscope. (B) Non-infection of control Sf9 cells. (C) Lysis of Sf9 cells was observed by light microscope after interaction for 3 h. Scale bar = 5 µm (left panel); 10 µm (right panel). (DOCX) [file ppat.1004288.s004.docx]

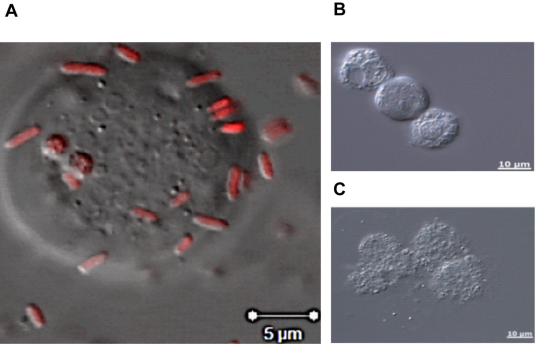


**Figure S4.** **Interaction of Sf9 insect cells and RFP-labeled *P. taiwanensis***. (A) RFP-labelled *P. taiwanensis* was incubated for 1 h with Sf9 cells and observed by confocal microscope. (B) Non-infection of control Sf9 cells. (C) Lysis of Sf9 cells was observed by light microscope after interaction for 3 h. Scale bar = 5 μm (left panel); 10 μm (right panel).
