# Supplementary material for: Characterization of an Insecticidal Toxin and Pathogenicity of Pseudomonas taiwanensis against Insects
Source: PLoS Pathog. 2014 Aug 21;10(8):e1004288. doi: 10.1371/journal.ppat.1004288 (PMC4140846; doi:10.1371/journal.ppat.1004288)
Supplement: Figure S5 — Determination of protease activity in P. taiwanensis . Proteolytic activity of the culture supernatant of P. taiwanensis in early (24 h), middle (36 h), and late (48 h) stationary phase was measured by azocasein substrate at 440 nm. (DOCX) [file ppat.1004288.s005.docx]

**
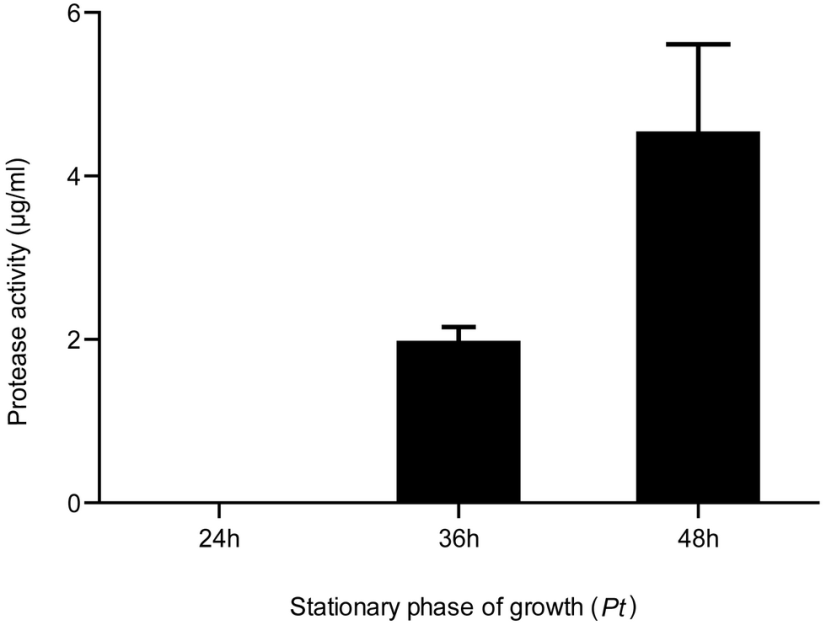
**

**Figure S5. Determination of protease activity in *P. taiwanensis*.** Proteolytic activity of the culture supernatant of *P. taiwanensis* in early (24 h), middle (36 h), and late (48 h) stationary phase was measured by azocasein substrate at 440 nm.
