# Supplementary material for: Characterization of an Insecticidal Toxin and Pathogenicity of Pseudomonas taiwanensis against Insects
Source: PLoS Pathog. 2014 Aug 21;10(8):e1004288. doi: 10.1371/journal.ppat.1004288 (PMC4140846; doi:10.1371/journal.ppat.1004288)
Supplement: Figure S6 — Comparison of hydrogen peroxide decomposition by wild-type and tccC mutant of P. taiwanensis . Degradation of hydrogen peroxide was measured in the culture medium at different time points. (DOCX) [file ppat.1004288.s006.docx]

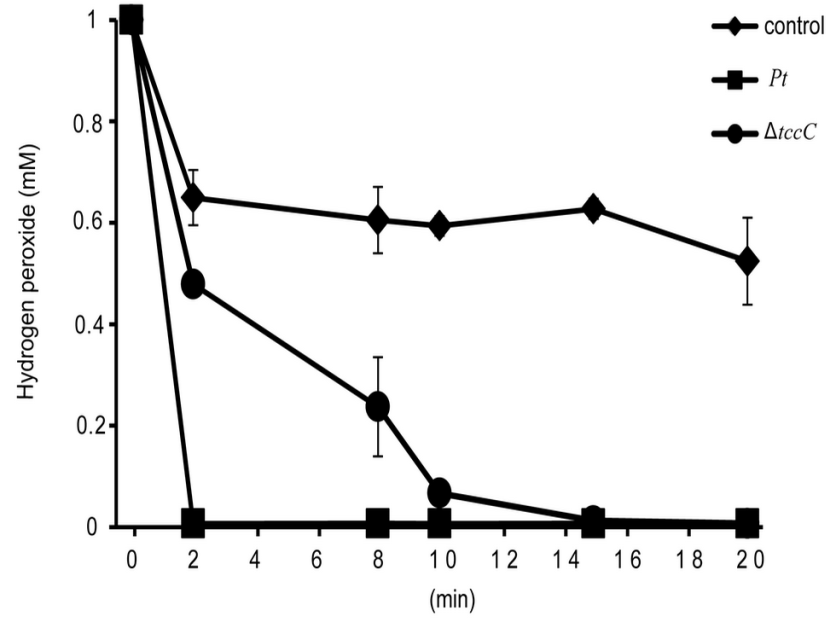


**Figure S6. Comparison of hydrogen peroxide decomposition by wild-type and *tccC* mutant of *P. taiwanensis*.** Degradation of hydrogen peroxide was measured in the culture medium at different time points.
