# Supplementary material for: Characterization of an Insecticidal Toxin and Pathogenicity of Pseudomonas taiwanensis against Insects
Source: PLoS Pathog. 2014 Aug 21;10(8):e1004288. doi: 10.1371/journal.ppat.1004288 (PMC4140846; doi:10.1371/journal.ppat.1004288)
Supplement: Figure S7 — TccC promotes glutamate uptake activity in P. taiwanensis . Wild-type and ΔtccC mutant P. taiwanensis cells were cultured in medium containing 250 µM of 15N-L-glutamate for 4 hours. Uptake activity of 15N-L-glutamate was defined as enrichment of 15N content in P. taiwanensis cells. The 15N enrichment was calculated as 15N abundance in labeled cells minus 15N abundance in non-labeled cells. The asterisk indicates a P value = 0.035 from triplet repeats, as determined by Student's t-test. (DOCX) [file ppat.1004288.s007.docx]

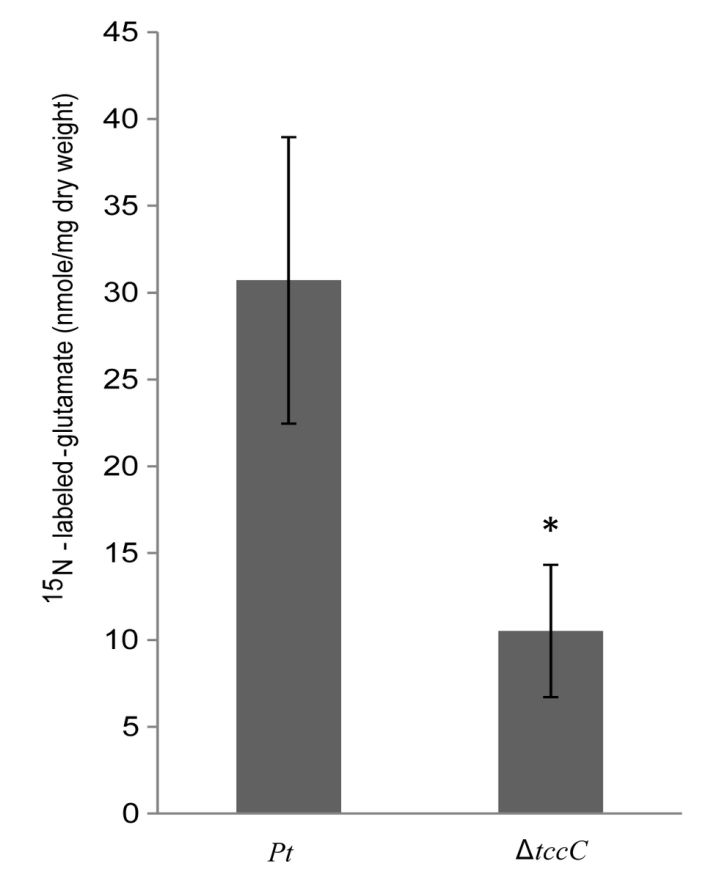


**Figure S7.** **TccC promotes glutamate uptake activity in *P. taiwanensis*.**

Wild-type and ΔtccC mutant *P. taiwanensis* cells were cultured in medium containing 250 μM of ^15^N-L-glutamate for 4 hours. Uptake activity of ^15^N-L-glutamate was defined as enrichment of ^15^N content in *P. taiwanensis* cells. The ^15^N enrichment was calculated as ^15^N abundance in labeled cells minus ^15^N abundance in non-labeled cells. The asterisk indicates a P value﹦0.035 from triplet repeats, as determined by Student's t test.
