# Supplementary material for: Characterization of an Insecticidal Toxin and Pathogenicity of Pseudomonas taiwanensis against Insects
Source: PLoS Pathog. 2014 Aug 21;10(8):e1004288. doi: 10.1371/journal.ppat.1004288 (PMC4140846; doi:10.1371/journal.ppat.1004288)
Supplement: Figure S8 — Survival of macrophages after treatment with wild-type or tccC mutant of P. taiwanensis in vitro. Macrophages incubated with wild-type or tccC mutant of P. taiwanensis (MOI = 1000) for 24 h and survival rates were detected by XTT assay. (DOCX) [file ppat.1004288.s008.docx]

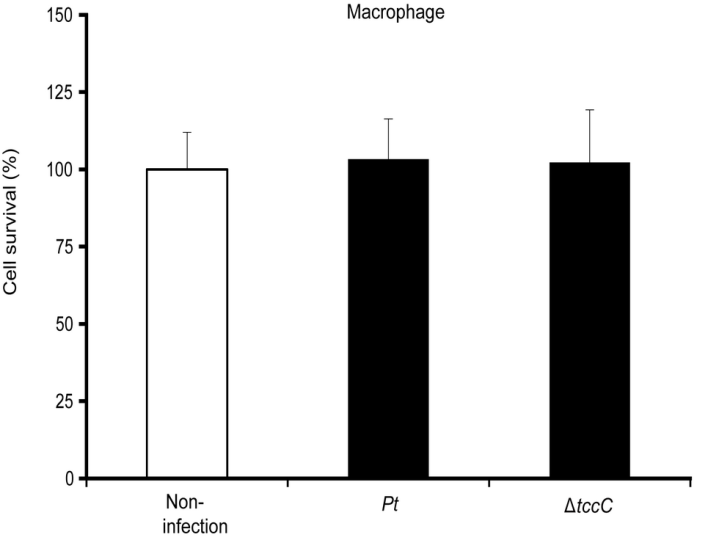


**Figure S8.** **Survival of macrophages after treatment with wild-type or *tccC* mutant of *P. taiwanensis* in vitro.** Macrophages incubated with wild-type or tccC mutant of *P. taiwanensis* (MOI = 1000) for 24 h and survival rates were detected by XTT assay.
