# Supplementary material for: Characterization of an Insecticidal Toxin and Pathogenicity of Pseudomonas taiwanensis against Insects
Source: PLoS Pathog. 2014 Aug 21;10(8):e1004288. doi: 10.1371/journal.ppat.1004288 (PMC4140846; doi:10.1371/journal.ppat.1004288)
Supplement: Figure S9 — Western blot analysis of cleavage of the C-terminal TccC fragment by immobilized metal affinity column. Overexpression of the whole length tccC gene in E. coli and P. taiwanensis by broad host vector pCPP30. Cleaved TccC was purified by immobilized metal affinity column and detected by TccC antibody. (DOCX) [file ppat.1004288.s009.docx]

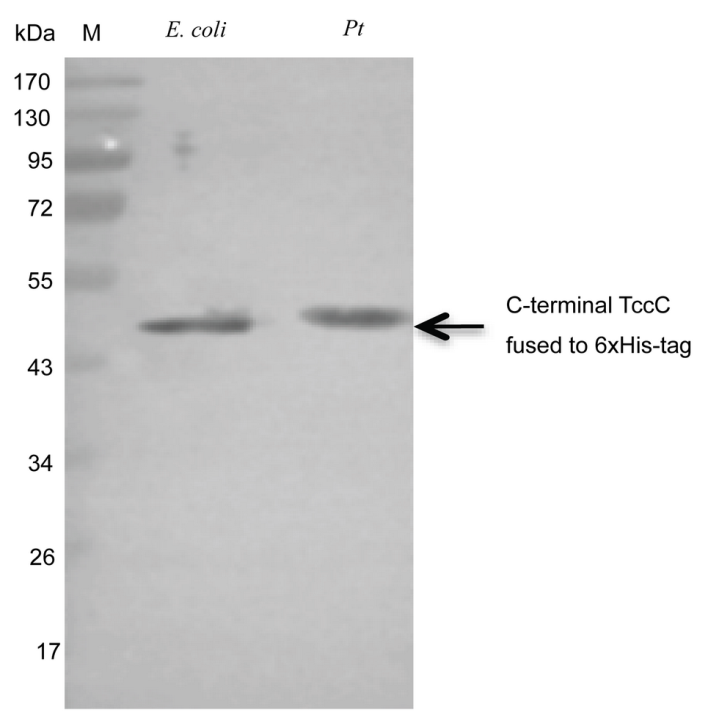


**Figure S9. Western blot analysis of cleavage of the C-terminal TccC fragement by immobilized metal affinity column.** Overexpression of the whole length *tccC* gene in *E. coli* and *P. taiwanensis* by broad host vector pCPP30. Cleaved TccC was purified by immobilized metal affinity column and detected by TccC antibody.
