# Supplementary material for: Characterization of an Insecticidal Toxin and Pathogenicity of Pseudomonas taiwanensis against Insects
Source: PLoS Pathog. 2014 Aug 21;10(8):e1004288. doi: 10.1371/journal.ppat.1004288 (PMC4140846; doi:10.1371/journal.ppat.1004288)
Supplement: Table S1 — Rough screening tests of mortality of insect larvae after oral infection with P. taiwanensis . P. taiwanensis was grown overnight and collected. Each bacteria fermentation (30 µl) was individually applied to 10 surfaces of 0.5×1 cm2 vegetable pieces for 30 larvae, which were used for feeding larvae and incubated at 25°C. Each infected larva was observed at day 5 after oral infection and the mortality rate was calculated. The two-tail student t-test was used to determine statistical significance. Each treatment (30 larvae) was repeated three times. The control was germ-free LB medium treatment. (DOCX) [file ppat.1004288.s010.docx]

**Table S1. Rough screening tests of mortality of insect larvae after oral infection with *P. taiwanensis*.** *P. taiwanensis* was grown overnight and collected. Each bacteria fermentation (30 µl) was individually applied to 10 surfaces of 0.5 × 1 cm^2^ vegetable pieces for 30 larvae, which were used for feeding larvae and incubated at 25°C. Each infected larva was observed at day 5 after oral infection and the mortality rate was calculated. The two-tail student t-test was used to determine statistical significance. Each treatment (30 larvae) was repeated three times. The control was germ-free LB medium treatment.

| **Treatment*^a^*** | **%Treated mortality(*n*)*^b^*** | **P value (two tailed)*^c^*** |
| --- | --- | --- |
| **Insect species** |  |  |
| *Plutella xylostella* | 23.3% | P<0.05 |
| *Spodoptera exigua* | 23.3% | P<0.05 |
| *Spodoptera litura* | 3.3% | P<0.05 |
| *Trichoplusia ni* | 20% | P<0.05 |
| *Helicoverpa armigera* Hubner | 0 | P<0.05 |
| Control | 0 | P<0.05 |
